# PBK, targeted by EVI1, promotes metastasis and confers cisplatin resistance through inducing autophagy in high-grade serous ovarian carcinoma

**DOI:** 10.1038/s41419-019-1415-6

**Published:** 2019-02-18

**Authors:** Hanlin Ma, Yingwei Li, Xiangxiang Wang, Huan Wu, Gonghua Qi, Rongrong Li, Ning Yang, Min Gao, Shi Yan, Cunzhong Yuan, Beihua Kong

**Affiliations:** 1 Department of Obstetrics and Gynecology, Qilu Hospital, Shandong University, 250012 Jinan, China; 2 Gynecologic Oncology Key Laboratory of Shandong Province, Qilu Hospital, Shandong University, 250012 Jinan, China; 3 Institute of Oncology, School of Medicine, Shandong University, 250012 Jinan, China

## Abstract

High-grade serous ovarian carcinoma (HGSOC) is the most lethal type of gynecologic malignancy. Chemoresistance is the main reason for the poor prognosis of HGSOC. PDZ-binding kinase (PBK) promotes the malignant progression of various carcinomas. However, the roles and clinical significance of PBK in HGSOC remain unclear. Here, we reported that PBK was overexpressed in HGSOC tissues and cell lines. High PBK expression was associated with a poor prognosis, metastasis, and cisplatin resistance of HGSOC. Overexpression of PBK promoted autophagy and enhanced cisplatin resistance via the ERK/mTOR signaling pathway. Further study showed that inhibition of autophagy by chloroquine or bafilomycin A1 reversed PBK-induced cisplatin resistance. Overexpression of PBK decreased ovarian cancer responsiveness to cisplatin treatment through inducing autophagy in vivo. We also demonstrated that the PBK inhibitor OTS514 augmented the growth inhibition effect of cisplatin in vitro and in vivo. Moreover, ecotropic viral integration site-1 (EVI1) could regulate PBK expression through directly targeting the PBK promoter region. In conclusion, high PBK expression was correlated with a poor prognosis, metastasis, and cisplatin resistance through promoting autophagy in HGSOC. PBK might be a promising target for the early diagnosis and individual treatment of ovarian cancer.

## Introduction

Ovarian cancer is the most lethal type of gynecologic malignancy^1^. In 2018, 22,240 new ovarian cancer cases and 14,070 ovarian cancer deaths are estimated to occur in the United States according to the American Cancer Society^2^. Ovarian cancer is conventionally treated with surgery and platinum/paclitaxel-based chemotherapy. High-grade serous ovarian carcinoma (HGSOC), the most common histological subtype, accounts for ~70% of all ovarian cancer cases^3^. The 5-year overall survival (OS) of HGSOC is between 35% and 40% due to primary treatment resistance in 15–25% of cases and the emergence of chemotherapy resistance in most of the remaining women^4^. However, the molecular mechanisms contributing to the chemotherapy resistance of HGSOC is obscure. It is necessary to elucidate the mechanisms of chemotherapy resistance and develop new target drugs.

PDZ-binding kinase (PBK), also known as T-LAK (lymphokine-activated killer T) cell-originated protein kinase (TOPK), was first cloned from the T-LAK cell subtraction cDNA fragment library^5^. PBK is a serine/threonine kinase belonging to the mitogen-activated protein kinase kinase (MAPKK) family^6^. PBK is rarely expressed in normal tissues except for fetal and germ cells but is highly trans-activated in various cancers, making it a promising molecular target for cancer screening and targeted therapy^7,8^. Many studies have indicated that high PBK expression is associated with a more aggressive phenotype in various cancers, including gastric, oral, glioma, lung, colon, colorectal, breast, prostate, and pancreatic cancers^9–20^. In epithelial ovarian cancer, high PBK expression is significantly associated with poor progression-free survival (PFS) and OS in early-stage cases. Additionally, the specific PBK inhibitors OTS514, OTS964, HI-TOPK-032, and SKLB-C05 shows strong growth-inhibitory effects in vitro and in vivo^14,21,22^. However, the functional mechanism of PBK in ovarian cancer remains unknown.

Autophagy is an evolutionarily conserved catabolic progress that involves the formation of double-membraned vesicles known as autophagosomes that degrade and recycle damaged proteins and old organelles in all eukaryotic cells^23^. Autophagy is also an adaptive process that is activated in response to various forms of stress, including nutrient depletion, virus infection, chemical drug stimulation, and hypoxia^24^. Excessive activated autophagy is considered the main reason contributing to chemoresistance in cancer therapy, and autophagy inhibition could promote paclitaxel/cisplatin-induced cell death^25–27^.

Recent studies have shown that miR-216b mediates the downregulation of PBK-enhanced chemosensitivity of colorectal cancer, and PBK inhibition could sensitize tumors to radiation^28,29^. Additionally, overexpression of PBK promotes the chemotherapeutic resistance to temozolomide, a first-line chemotherapy drug in glioma^30^. PBK could interact with the p53 DNA-binding domain and promote cancer cell resistance to doxorubicin treatment through p21 inhibition^31^. PBK expression is higher in cisplatin-resistant A2780/DDP cells than in sensitive A2780 cells, indicating that PBK might participate in the chemoresistance of ovarian cancer^32^. PBK is associated with several signal transduction pathways involved in the regulation of cellular autophagy, including MAPK, PI3K/AKT, and mTOR, suggesting that PBK might participate in autophagy regulation^9,13^.

In this study, we aim to illuminate the functions of PBK in the chemoresistance and autophagy of HGSOC and further investigate the underlying mechanisms in vitro and in vivo. Additionally, we try to elucidate the regulatory mechanism of PBK in HGSOC.

## Results

### Elevated PBK expression correlates with the poor prognosis and chemoresistance of HGSOC

**Fig. 1:**
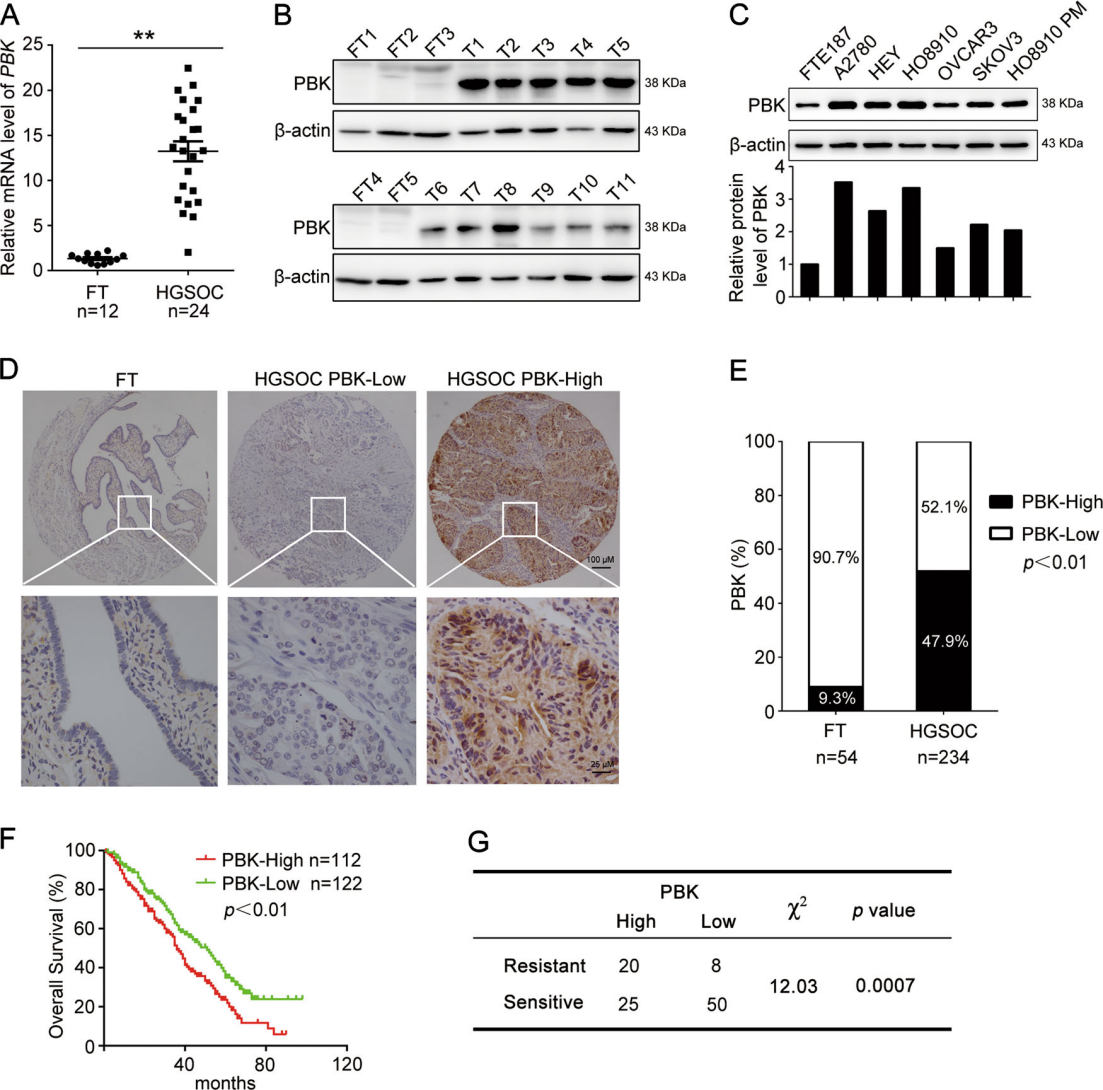
PBK is correlated with poor prognosis and cisplatin resistance in HGSOC. **a** qPCR analysis of the mRNA level of PBK in high-grade serous ovarian cancer (HGSOC) tissues and normal fallopian tube (FT) tissues (FT, *n* = 12; HGSOC, *n* = 24). **b** Western blot analysis of the protein level of PBK in HGSOC tissues and FT tissues (FT, FT1-FT5; HGSOC, T1-T11; uncut bands were shown in Supplementary Figure S1A). **c** Western blot analysis of the protein level of PBK in ovarian cancer cell lines (A2780, HEY, HO8910, OVCAR3, SKOV3, HO8910PM) and normal fallopian tube epithelial cells (FTE187). **d** Representative images of IHC staining of PBK in HGSOC tissues and FT tissues (upper, ×40 ; lower, ×400). **e** The high and low expression rate of PBK in 54 FTE tissues and 234 HGSOC tissues. **f** Overall survival (OS) curves of HGSOC patients with high or low PBK expression level in our cohort. **g** Correlation between PBK expression and cisplatin resistance in HGSOC patients (data are mean ± SEM, **p < 0.01)

We first examined PBK expression levels in HGSOC and fallopian tube (FT) tissues, and the results showed that the mRNA and protein levels of PBK were significantly higher in HGSOC tissues than in normal FT tissues (Fig. 1a, b). The results from datasets within Oncomine and GEPIA also showed that PBK was overexpressed in ovarian cancer samples compared with the peritoneum or ovarian surface epithelium tissues (Supplementary Fig. S2A–E). Furthermore, the expression levels of PBK in most ovarian cancer cell lines (5/6) were obviously higher than those in FTE187 cells (Fig. 1c). These results showed that the expression of PBK was extensively high in HGSOC, indicating a possible role of PBK in ovarian cancer.

To assess the expression pattern and clinical significance of PBK in HGSOC, we performed immunohistochemistry (IHC) to measure PBK expression in tissue microarrays (HGSOC, *n* = 234; FT, *n* = 54). We divided HGSOC patients into the low PBK expression group (PBK-low) and high PBK expression group (PBK-high) based on IHC results (Fig. 1d). There was a significantly higher expression of PBK in HGSOC tissues (47.9%, 112/234 cases) than in FT tissues (9.3%, 5/54 cases) (Fig. 1e). Clinicopathological characteristic analysis showed that PBK expression was correlated with lymph node metastasis but not with other features (Table 1). Kaplan–Meier survival curves showed that HGSOC patients with high PBK expression exhibited a significantly shorter OS than patients with low PBK expression (Fig. 1f). The mRNA expression level of PBK using the Kaplan–Meier plotter also showed significantly shorter OS and PFS in patients with high PBK expression (Supplementary Fig. S2F–G). Furthermore, the expression of PBK in cisplatin-resistant patients was significantly higher than that in sensitive patients (Fig. 1g), suggesting that PBK played a significant role in the chemoresistance of HGSOC.

**Table 1:** Correlation between PBK expression and clinicopathological characteristics

Clinicopathological features		PBK expression		
		Low expression	High expression	*p* value
Ages (years)	<56	36	42	0.1952
	≥56	86	70	
FIGO staging	I + II	28	18	0.1977
	III + IV	93	92	
CA125 (U/ml)	<600	45	47	0.5511
	≥600	60	53	
Lymph node metastasis	Positive	30	45	0.0069
	Negative	42	25	
Omentum metastasis	Positive	79	74	0.6922
	Negative	36	30	

### PBK confers ovarian cancer cells with cisplatin resistance

**Fig. 2:**
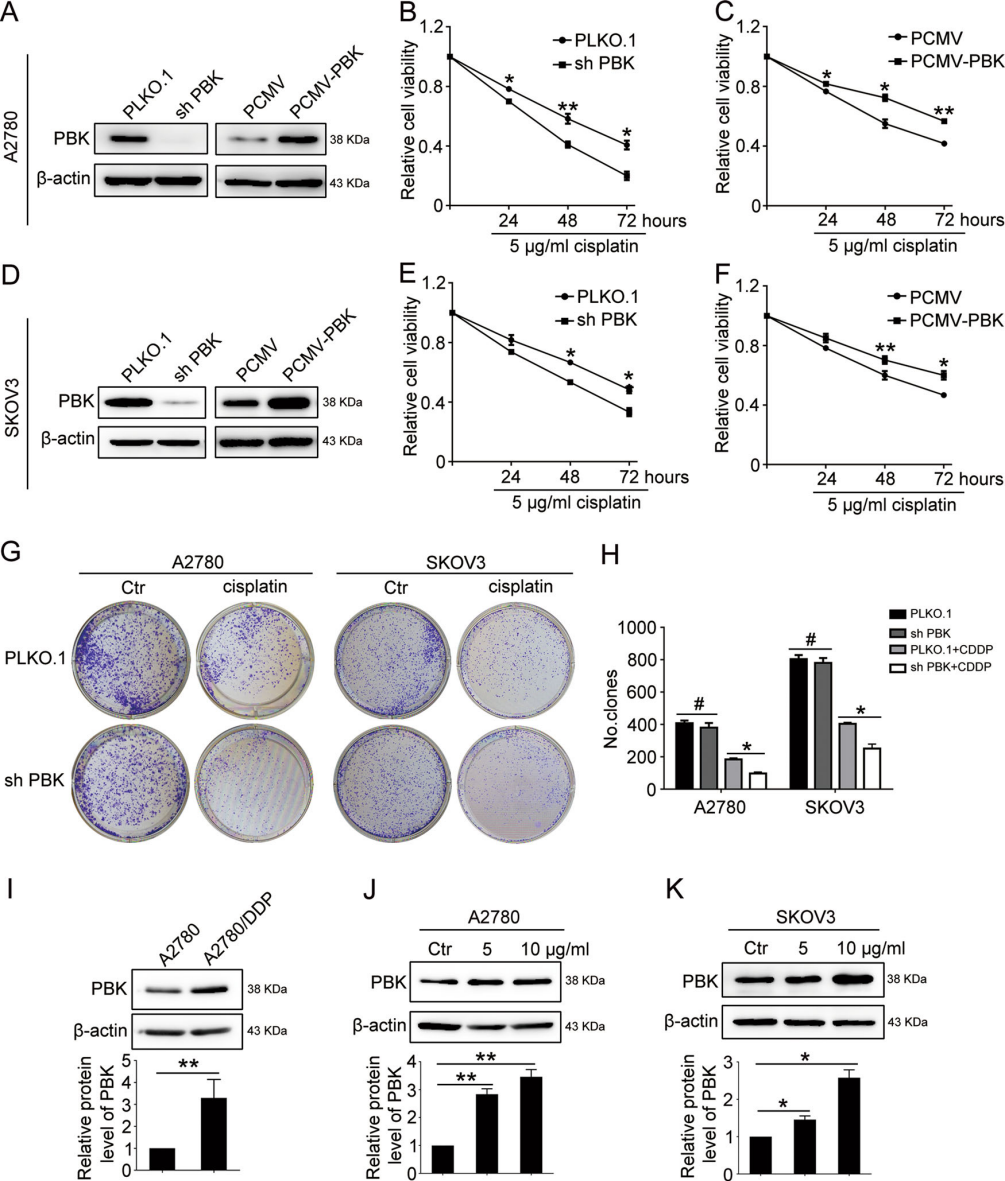
PBK confers cisplatin resistance in ovarian cancer cell lines. A2780 and SKOV3 cells were stably transfected with PLKO.1, PBK shRNA (sh-PBK), PCMV. and PCMV-PBK, then treated with 5 μg/ml cisplatin (CDDP) for 24, 48, and 72 h. Protein levels of PBK and β-actin were analyzed by western blot in A2780 (**a**) or SKOV3 (**d**) cells. Cell viability was measured using CCK8 in A2780 (**b**, **c**) or SKOV3 (**e**, **f**) cells. **g** Clonogenic assay was performed to assess the colony formation efficiency of A2780 and SKOV3 cells stably transfected with PLKO.1 and PBK shRNA with or without 5 μg/ml cisplatin treatment. **h** Quantification of the number of clones in **g**. **i** Western blot analysis of PBK protein level in A2780 and A2780/DDP cells. Western blot analysis of PBK protein level in A2780 (**j**) and SKOV3 (**k**) cells treated with 5 or 10 μg/ml cisplatin for 24 h (data are mean ± SEM, ^#^*p* > 0.05, *p < 0.05, **p *<* 0.01, *n* = 3)

A2780 and SKOV3 cells stably transfected with PCMV-PBK (PCMV-PBK), PBK shRNA (sh-PBK), and negative controls (PCMV, PLKO.1, respectively) were exposed to cisplatin (5 μg/ml) for different times (24, 48, 72 h). The Cell Counting Kit-8 (CCK8) assay showed that cells with sh-PBK exhibited higher sensitivity to cisplatin cytotoxicity than the control group. Correspondingly, the overexpression of PBK conferred ovarian cancer cells with cisplatin resistance (Fig. 2a–f). The clonogenic assay also confirmed the cisplatin-resistant role of PBK (Fig. 2g, h). We also performed cell proliferation and clonogenic assays in cisplatin-resistant A2780/DDP cells in which cisplatin (10 μg/ml) was ineffective to repress cell growth (Supplementary Fig. S3). The results showed that the knockdown of PBK significantly increased cisplatin sensitivity in A2780/DDP cells, further justifying that PBK conferred cisplatin resistance. Additionally, the expression of PBK was elevated in cisplatin-resistant A2780/DDP cells compared with that in cisplatin-sensitive A2780 cells (Fig. 2i). There was also a dose-dependent increase in PBK expression after the cells were exposed to cisplatin (Fig. 2j, k). Therefore, increased expression of PBK contributed to the cisplatin resistance of ovarian cancer.

### PBK promotes the migration and invasion of ovarian cancer cells

**Fig. 3:**
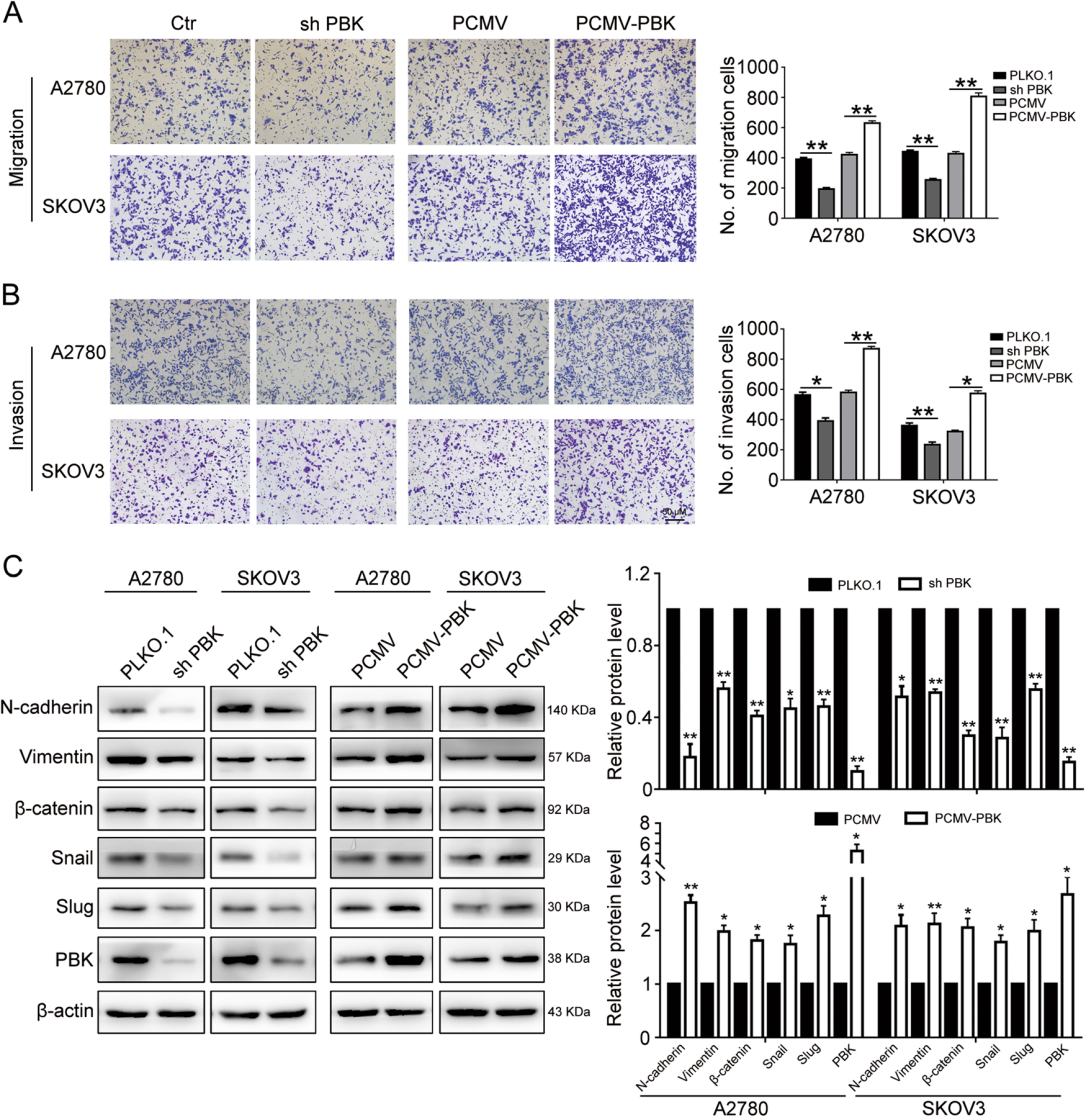
PBK promotes migration and invasion of ovarian cancer cells. **a**, **b** Transwell assay was performed to determinate the effects of PBK knockdown or overexpression on migration and invasion of A2780 and SKOV3 cells. **c** EMT-related markers were detected by western blot in A2780 and SKOV3 cells with PBK knockdown or overexpression (data are mean ± SEM, *p < 0.05, **p *<* 0.01, *n* = 3)

Considering that PBK expression was associated with lymph node metastasis in the patient cohort, we hypothesized that PBK participated in the metastasis of ovarian cancer cell lines. The Transwell assay showed that the knockdown of PBK dramatically impaired the migration and invasion abilities of A2780 and SKOV3 cells, while the overexpression of PBK enhanced these abilities (Fig. 3a, b). To exclude the influence of proliferation on metastasis, A2780 and SKOV3 cells were treated with mitomycin C (10 μg/ml) for 2 h to inhibit cell proliferation prior to the Transwell assay, and the results showed that the knockdown of PBK dramatically impaired the invasion abilities of A2780 and SKOV3 cells with mitomycin C treatment (Supplementary Fig. S4), consistent with previous results. Western blotting was conducted to measure epithelial–mesenchymal transition (EMT) marker expression. The results indicated that PBK knockdown downregulated the mesenchymal markers N-cadherin, vimentin, β-catenin, Snail, and slug. Correspondingly, PBK overexpression increased the expression of these mesenchymal markers (Fig. 3c). These results indicated that PBK promoted ovarian cancer cell metastasis in vitro.

### NGS assay of DEGs and related signaling pathways

To illuminate the underlying pathways involved in PBK-induced chemoresistance and metastasis, next-generation sequencing (NGS) was conducted to investigate the gene expression profiles of PBK knockdown (si PBK) A2780 cells and control cells (Ctr). The high-throughput sequencing identified 1379 differentially expressed genes (DEGs; [log2(FoldChange)] > 1 and *p* < 0.05) in A2780 cells after transfection with PBK siRNA for 48 h, including 417 upregulated genes and 962 downregulated genes (Supplementary Fig. S5A). Kyoto Encyclopedia of Genes and Genomes (KEGG) was performed to demonstrate the signaling pathways correlated with PBK expression. The results showed that the MAPK, Rap1, PI3K–AKT, and Hippo signaling pathways were strongly enriched in the PBK knockdown group (Supplementary Fig. S5B–C). qPCR was performed to verify the downregulation of representative DEGs involved in the MAPK, Rap1, and PI3K–AKT signaling pathways (Supplementary Fig. S5D). These signaling pathways all participated in the proliferation, invasion, and chemoresistance of ovarian cancer. We speculated that these pathways might be involved in the PBK-induced chemoresistance and metastasis of ovarian cancer.

### PBK promotes autophagy through the ERK/mTOR axis

**Fig. 4:**
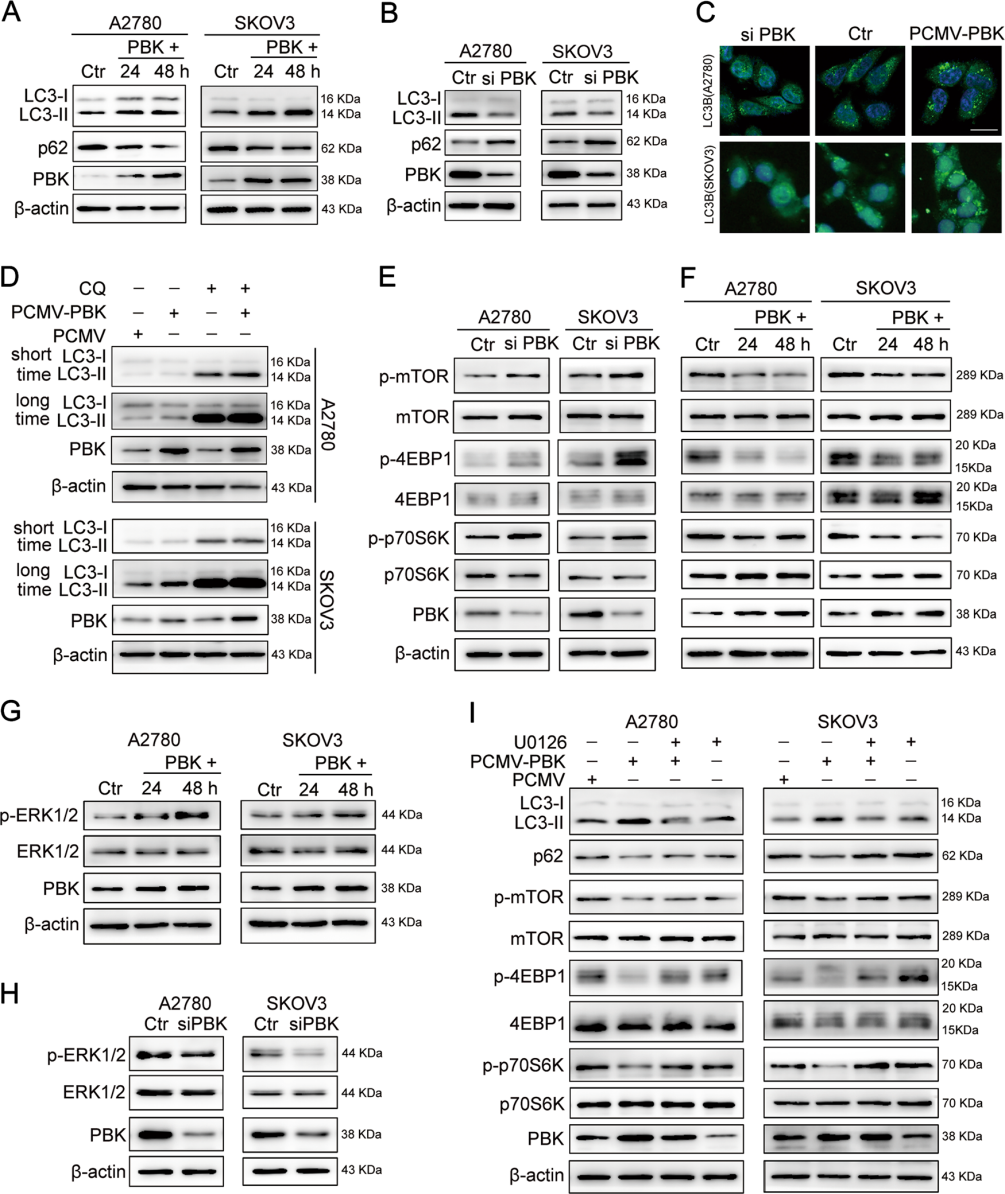
PBK promotion of autophagy through ERK/mTOR signal pathway. **a** Western blot analysis of protein levels of LC3-I, LC3-II, p62, PBK, and β-actin in A2780 or SKOV3 cells transfected with PCMV (Ctr) or PCMV-PBK (PBK+) for 24 or 48 h. **b** Western blot analysis of protein levels of LC3-I, LC3-II, p62, PBK, and β-actin in A2780 or SKOV3 cells transfected with negative control RNA (Ctr) or PBK siRNA (si PBK) for 48 h. **c** Immunofluorescence staining of LC3B in A2780 and SKOV3 cells transfected with PBK siRNA (si PBK), negative control RNA (Ctr), PCMV (Ctr), and PCMV-PBK for 48 h. Scale bar: 16 µm. **d** Western blot analysis of protein levels of LC3-I, LC3-II, PBK, and β-actin in A2780 or SKOV3 cells transfected with PCMV or PCMV-PBK for 24 h with or without CQ (50 μM) treatment. **e** Western blot analysis of protein levels of p-mTOR, mTOR, p-4EBP1, 4EBP1, p-p70S6K, p70S6K, PBK, and β-actin in A2780 or SKOV3 cells transfected with negative control RNA (Ctr) or PBK siRNA (si PBK) for 48 h. **f** Western blot analysis of protein levels of p-mTOR, mTOR, p-4EBP1, 4EBP1, p-p70S6K, p70S6K, PBK, and β-actin in A2780 or SKOV3 cells transfected with PCMV (Ctr) or PCMV-PBK (PBK+) for 24 or 48 h. **g** Western blot analysis of protein levels of p-ERK1/2, ERK1/2, PBK, and β-actin in A2780 or SKOV3 cells transfected with PCMV (Ctr) or PCMV-PBK (PBK+) for 24 or 48 h. **h** Western blot analysis of protein levels of p-ERK1/2, ERK1/2, PBK, and β-actin in A2780 or SKOV3 cells transfected with negative control RNA (Ctr) or PBK siRNA (si PBK) for 48 h. **i** A2780 or SKOV3 cells were transfected with PCMV or PCMV-PBK for 24 h, then cultured with or without 10 μM U0126 for 24 h. Protein levels of LC3-I, LC3-II, p62, p-mTOR, mTOR, p-4EBP1, 4EBP1, p-p70S6K, p70S6K, PBK, and β-actin were measured by western blot. Quantification of relative protein expression levels was shown in Supplementary Figures S6 and S7

Considering that PBK was involved in the regulation of the MAPK and PI3K–AKT signaling pathway, which mediates the progression of autophagy, we investigated the role of PBK in the autophagy of ovarian cancer cells. Transient overexpression of PBK for 24 and 48 h increased the level of autophagic protein microtubule-associated protein 1 light chain 3- II (LC3-II) and decreased the level of autophagy substrate p62 compared with those in the control groups (Fig. 4a). Knocking down PBK showed opposite results compared with that in the negative control groups in A2780 and SKOV3 cells (Fig. 4b). Consistently, the immunofluorescence assay showed a decreased proportion of cells containing LC3B puncta (>5) in cells transfected with PBK siRNA and increased proportion in cells with PCMV-PBK transfection (Fig. 4c). The upregulated LC3-II protein level evoked by PBK could be either ascribed to enhancing the initial autophagy pathway or blocking lysosomal degradation. To address this concern, chloroquine (CQ) was used to block autophagy flux. PBK could elevate LC3-II accumulation in the presence of CQ (Fig. 4d). These data indicated that PBK promoted autophagy flow in ovarian cancer cells. Additionally, the overexpression of PBK increased the phosphorylation of 4E-binding protein 1 (4EBP1), p70 S6 kinase (p70S6K), and mammalian target of rapamycin (mTOR), whereas PBK knockdown decreased the phosphorylation of these proteins (Fig. 4e, f). Thus, the overexpression of PBK activated ovarian cancer cell autophagy through suppressing the mTOR signaling pathway.

PBK, an MAPKK-like serine/threonine protein kinase, could function as the upstream kinase of p38/ERK signaling. However, PBK acts on different substrates in different cancer cells^5,11^. Until now, there is no report about the function of PBK in ERK1/2 phosphorylation in ovarian cancer. Our results showed that the overexpression of PBK increased the phosphorylation of ERK1/2, while the knockdown of PBK decreased the phosphorylation of ERK1/2 (Fig. 4g, h). Based on ERK inactivating 4EBP1 and p70S6K through inhibiting the activation of the mTOR complex 1 (ref. ^33^), we speculated that PBK promoted autophagy through the ERK/mTOR axis. As shown in Fig. 4i, the ERK inhibitor U0126 significantly impaired LC3-II accumulation and p62 downregulation induced by PBK. Downregulation of the phosphorylation of 4EBP1, p70S6K, and mTOR caused by PBK was attenuated as well. Additionally, the overexpression of PBK failed to promote ovarian cancer cell autophagy with mTOR or ERK1/2 knockdown, and the restoration of mTOR or ERK1/2 expression could partly rescue the autophagy blocked by knockdown of mTOR or ERK1/2 in A2780 and SKOV3 cells (Supplementary Fig. S8). These results indicated that PBK promotion of autophagy depended on the ERK/mTOR axis.

### PBK promotes cisplatin resistance through inducing autophagy

**Fig. 5:**
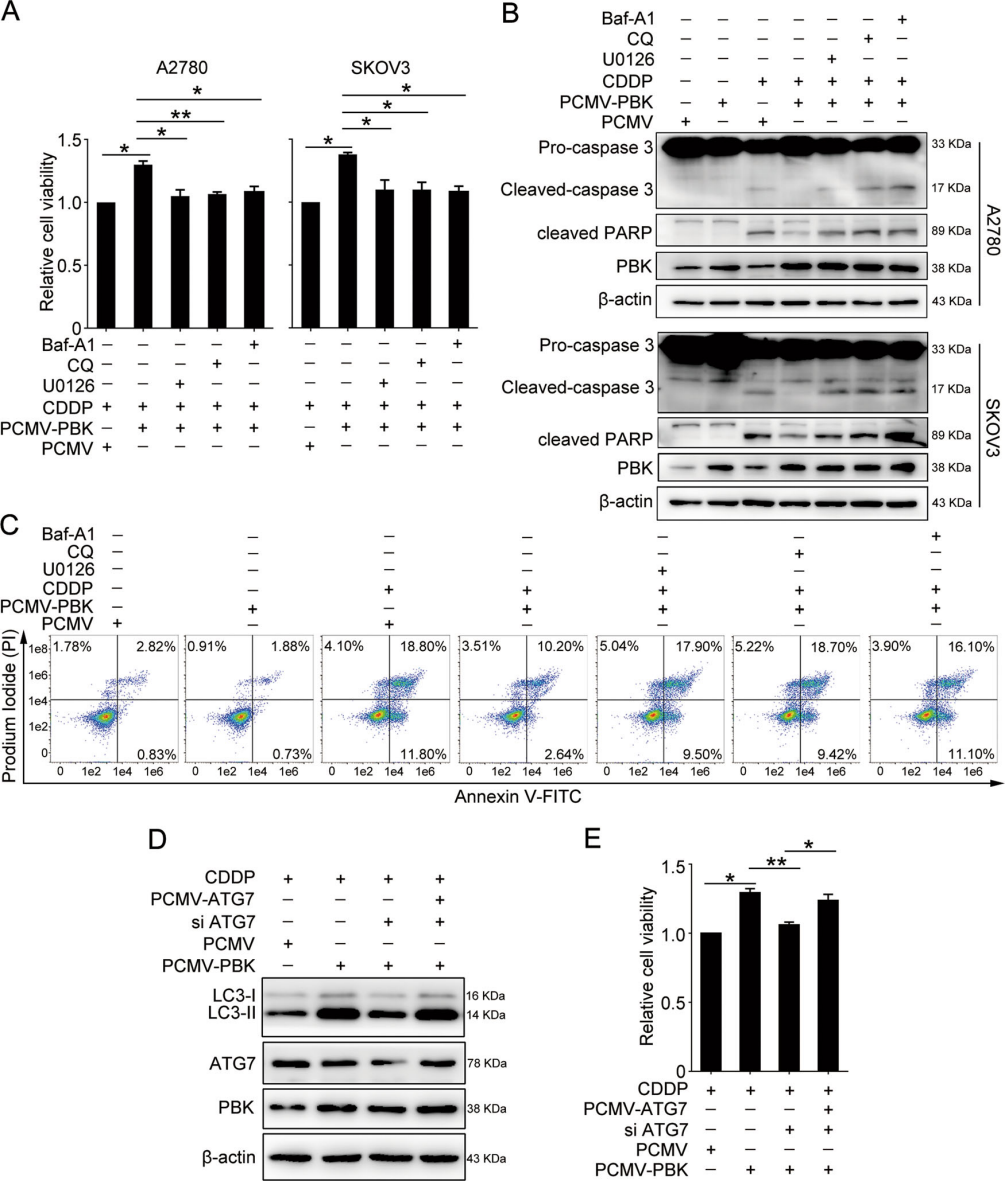
PBK confers cisplatin resistance through autophagy and ERK/mTOR axis. Cells were transfected with PCMV or PCMV-PBK for 24 h, then cultured with or without CDDP (5 μg/ml), U0126 (10 μM), CQ (50 μM), and Baf A1 (50 nM) for 24 h. **a** The CCK8 assay was used to determine the relative cell viability in A2780 and SKOV3 cells. **b** Western blot analysis of protein levels of cleaved-caspase-3, cleaved PARP, PBK, and β-actin in A2780 and SKOV3 cells. Quantification of relative protein expression levels was shown in Supplementary Figure S9. **c** The proportion of apoptotic A2780 cells were determined by AnnexinV-FITC/PI staining and flow cytometry. A2780 cells were transfected with PCMV, PCMV-PBK, PCMV-ATG7, or ATG7 siRNA (si ATG7) for 24 h, followed by 5 μg/ml cisplatin treatment for 24 h. **d** Western blot analysis of protein levels of LC3-I, LC3-II, ATG7, PBK, and β-actin. **e** The CCK8 assay was performed to detect the relative cell viability (data are mean ± SEM, *p < 0.05, **p < 0.01, *n* = 3)

We next determined whether the overexpression of PBK could reverse cisplatin-induced cell death in ovarian cancer cells. A2780 and SKOV3 cells were transfected with PCMV-PBK for 24 h, followed by 5 μg/ml of cisplatin treatment. Overexpression of PBK enabled the resistance to cisplatin lethality compared with PCMV transfection. However, blocking autophagy using bafilomycin A1 (Baf A1) or CQ abrogated this effect. The same effect was observed using the specific ERK inhibitor U0126 (Fig. 5a). Additionally, the overexpression of PBK reduced the levels of cleaved PARP and cleaved-caspase-3 induced by cisplatin treatment compared with PCMV transfection. Consistently, inhibition of autophagy or the ERK pathway could counteract the function of PBK overexpression (Fig. 5b). Flow cytometry results confirmed the reverse effects of autophagy and ERK inhibition on cisplatin-induced apoptosis in ovarian cancer cells (Fig. 5c). A rescue experiment was also performed to further prove the function of autophagy in mediating PBK-induced cisplatin resistance. The results showed that the knockdown of autophagy related 7 (ATG7), an essential factor in autophagy progression^34,35^, impaired PBK overexpression-induced cisplatin resistance, while the restoration of ATG7 expression could partly rescue the chemoresistance role of PBK in A2780 cells (Fig. 5d, e). Our data suggested that PBK exerted a cisplatin-resistant role through promoting autophagy.

### PBK inhibition enhances the lethal effect of cisplatin in vitro and in vivo

**Fig. 6:**
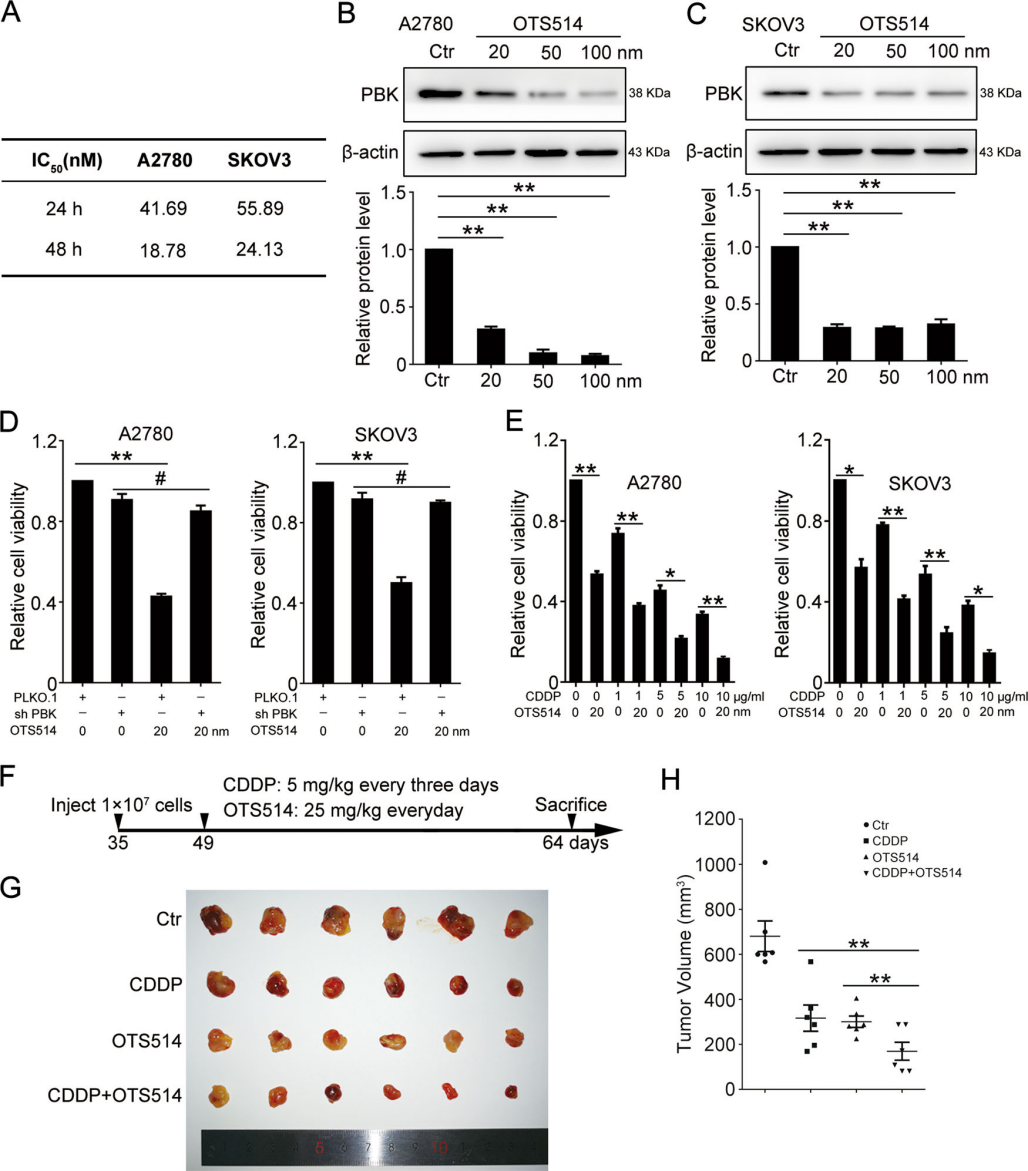
PBK inhibitor enhances the lethality effect of cisplatin. **a** The IC_50_ values of A2780 and SKOV3 treated with OTS514 for 48 h. Western blot analysis of protein levels of PBK and β-actin in A2780 (**b**) and SKOV3 (**c**) cells treated with 20, 50, 100 nM OTS514 for 48 h. **d** The CCK8 assay determination of relative cell viability in PLKO.1 and sh-PBK A2780 cells treated with or without 20 nM OTS514 for 48 h. **e** CCK8 assay determination of relative cell viability in A2780 and SKOV3 cells treated with or without 20 nM OTS514 and 1, 5, 10 μg/ml CDDP for 48 h (data are mean ± SEM, *p < 0.05, **p < 0.01, *n* = 3). **f** Experimental design of experimental protocols in BALB/c nude mice. Thirty-five-day-old mice were subcutaneously injected with A2780 cells. When the tumor volumes reached 50–200 mm^3^ at day 49, tumor-bearing mice then received intraperitoneal injection of CDDP (5 mg/kg, every 3 days) or/and oral administration of OTS514 (25 mg/kg, every day). Fifteen days after treatment, the mice were sacrificed to determine tumor volumes and were photographed. **g** Tumors from each group were shown. **h** The tumor volumes of each group (data are mean ± SEM, *p < 0.05, **p < 0.01, *n* = 6)

A previous study reported that the PBK inhibitors OTS514 and OTS964 showed a strong growth-inhibitory effect against ovarian cancer cells in vitro and in vivo^21^. We wondered whether PBK inhibition could enhance the lethal effect of cisplatin. Similar to previous results, OTS514 could effectively inhibit ovarian cancer cell proliferation at 24 and 48 h (Supplementary Fig. S10). The IC_50_ values of OTS514 for A2780 and SKOV3 cell lines were 18.78 and 24.13 nm, respectively (Fig. 6a). As shown in Fig. 6b, c, OTS514 (20 nm) treatment for 48 h significantly decreased the PBK protein level in A2780 and SKOV3 cell lines. OTS514 failed to inhibit ovarian cancer cell proliferation in PBK-knockdown cells (Fig. 6d), suggesting that OTS514 exerted a growth-inhibitory effect dependent on PBK expression. Additionally, OTS514 could effectively contribute to the growth-inhibitory effect of cisplatin in vitro and in vivo (Fig. 6e–h). Our results indicated the bright prospect of combining a PBK inhibitor with cisplatin for ovarian cancer therapy.

### PBK promotes ovarian cancer chemoresistance through autophagy in vivo

**Fig. 7:**
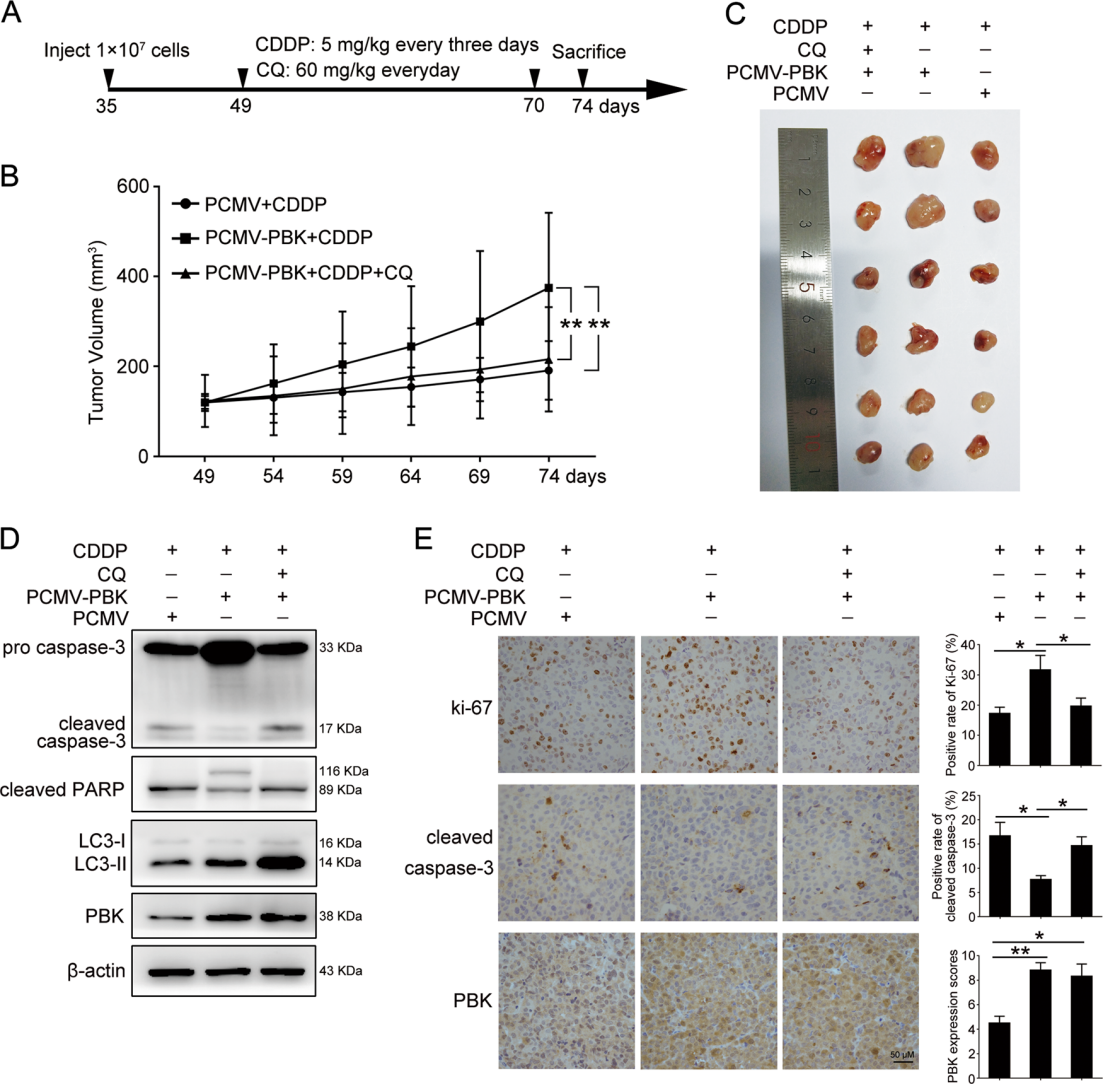
PBK promotes cisplatin resistance through autophagy in xenograft. **a** Experimental design of experimental protocols in BALB/c nude mice. Thirty-five-day-old mice were subcutaneously injected with cells stably transfected with PCMV-PBK or PCMV. When the tumor volumes reached 50–200 mm^3^ at day 49, tumor-bearing mice then received intraperitoneal injection of CDDP (5 mg/kg, every 3 days) or/and CQ (60 mg/kg, every day), respectively. The volumes of tumor were measured every 5 days using a vernier caliper. Twenty-five days after injection, the mice were sacrificed to determine tumor volumes and were photographed. **b** The tumor volumes of each group. **c** Tumors from each group were shown. **d** Western blot analysis of protein levels of cleaved-caspase-3, cleaved PARP, LC3-I, LC3-II, PBK, and β-actin in tumor tissues. Quantification of relative protein expression levels was shown in Supplementary Figure S11D. **e** Representative images of IHC staining of Ki-67, cleaved-caspase-3, and PBK in tumor tissues. Scale bar: 50 µm (data are mean ± SEM, *p < 0.05, **p < 0.01, *n* = 6)

Next, we investigated whether elevated PBK expression affected the tumor response to cisplatin treatment in vivo. PCMV-PBK or control PCMV cells were subcutaneously injected into the left armpit of female BALB/c nude mice (PCMV-PBK, *n* = 12; PCMV, *n* = 6) to generate a xenograft model, followed by intraperitoneal injection of cisplatin or/and CQ (Fig. 7a). Upon cisplatin treatment, the tumor volumes were significantly higher in mice injected with PCMV-PBK cells than in those injected with PCMV cells, and CQ reversed the effect of PBK overexpression (Fig. 7b, c). Consistently, the overexpression of PBK inhibited cell apoptosis induced by cisplatin, an effect that could also be counteracted by CQ administration (Fig. 7d, e). Additionally, subcutaneous transplantation of the PBK-knockdown A2780 cells into athymic mice led to increased cisplatin sensitivity (Supplementary Fig. S11A–C). Our findings indicated that PBK could confer ovarian cancer cells with cisplatin resistance in vivo.

### PBK is transcriptionally regulated by EVI1

**Fig. 8:**
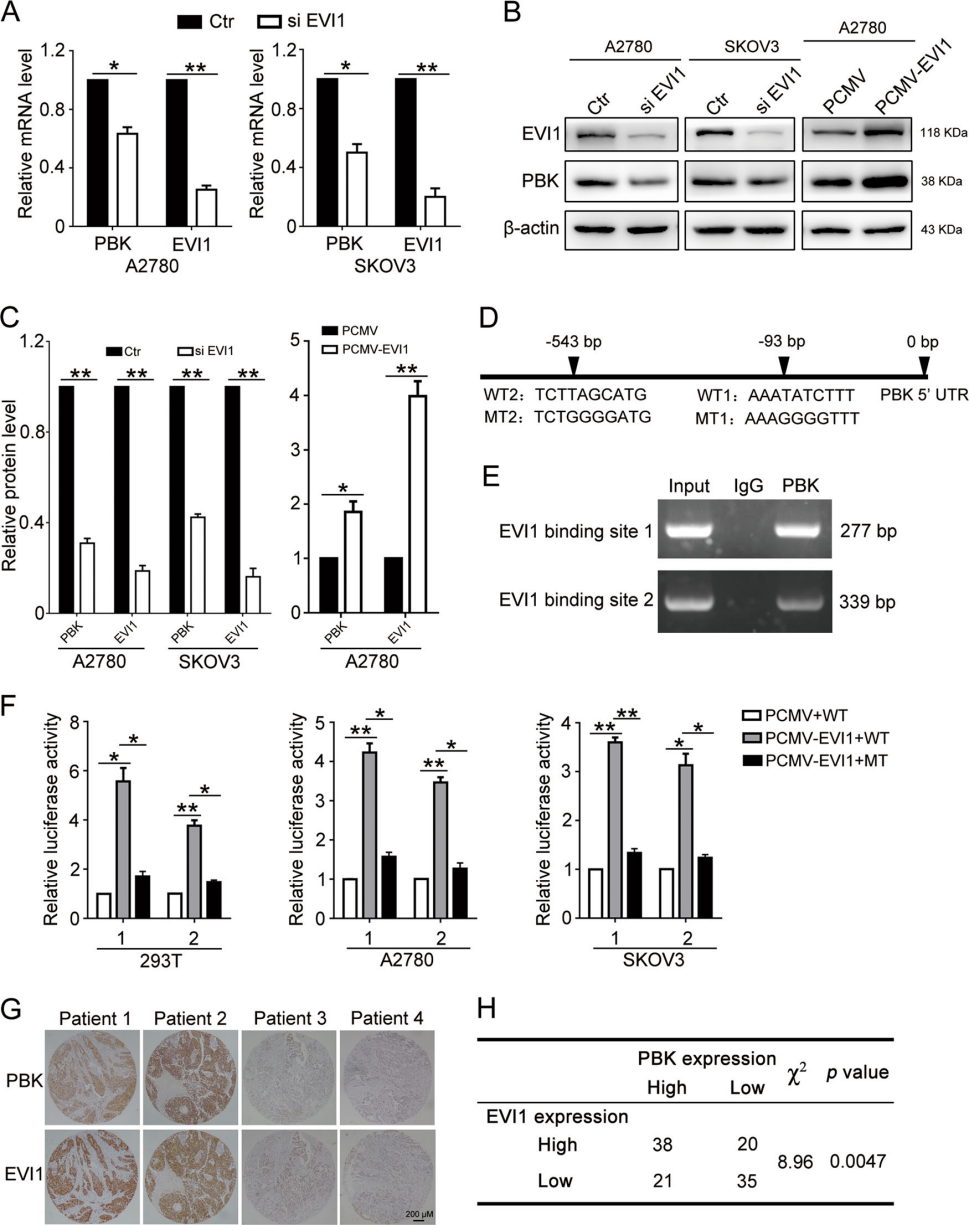
EVI1 directly regulates PBK expression through binding to the promoter of PBK. **a** qPCR analysis of the mRNA level of PBK and EVI1 in A2780 and SKOV3 cells transfected with EVI1 siRNA (si PBK) or negative control siRNA (Ctr) for 48 h. **b** Western blot analysis of protein levels of EVI1, PBK, and β-actin in A2780 and SKOV3 cells transfected with EVI1 siRNA (si PBK), negative control siRNA (Ctr), PCMV or PCMV-EVI1 for 48 h. **c** Quantification of relative protein expression levels in **b**. **d** Schematic representation of the potential EVI1-binding sites on PBK promoter region predicted by Consite (http://consite.genereg.net/) and JASPAR (http://jaspar.binf.ku.dk/). **e** Validation of the exact EVI1-binding site at the promoter of PBK examined by ChIP-PCR. **f** PCMV or PCMV-EVI1 plasmids were co-transfected into HEK293T, A2780, and SKOV3 cells with PBK promoter WT or MT plasmids for 24 h and luciferase activity were measured. **g** Representative images of IHC staining of EVI1 and PBK in HGSOC tissues. Scale bar: 200 µm. **h** Correlation between EVI1 and PBK expression in HGSOC patients (data are mean ± SEM, *p < 0.05, **p *<* 0.01, *n* = 3)

Finally, we tried to determine the regulatory mechanism of PBK in ovarian cancers. We searched for potential transcription factors that target the PBK promoter using the Consite and JASPAR online databases, and two ecotropic viral integration site-1 (EVI1)-binding sites were identified on the promoter of PBK. Knockdown of EVI1 decreased the mRNA and protein levels of PBK, while the overexpression of EVI1 increased the PBK levels (Fig. 8a–c). These results suggested that EVI1 regulated PBK expression. To confirm that PBK is a direct target of EVI1, we then introduced the wild-type PBK promoter region (WT1 and WT2) and corresponding mutant counterparts (MT1 and MT2) into the pGL4.26 vector (Fig. 8d). These vectors were then transfected into HEK293T, A2780, and SKOV3 cells with or without EVI1 expression. The results showed that EVI1 overexpression increased the luciferase activity in cells transfected with the wild-type promoter of PBK but decreased in cells with the mutant promoter of PBK (Fig. 8f). Additionally, ChIP-PCR was performed in A2780 cells to confirm that EVI1 could trans-activate PBK (Fig. 8e). We further performed IHC staining for PBK and EVI1 in tissue microarrays of 116 cases of HGSOC. The results demonstrated that the expression of PBK was positively correlated with EVI1 (Fig. 8g, h). Thus, EVI1 could regulate PBK transcription through directly targeting the PBK promoter in ovarian cancer cells.

## Discussion

Platinum/paclitaxel-based chemotherapy is the first-line treatment for ovarian cancer. Cisplatin resistance is the major reason for the failure of ovarian cancer treatment. However, the underlying mechanisms of cisplatin resistance remain obscure. Thus, it is of enormous clinical significance to understand the mechanisms of cisplatin resistance and develop new target drugs. PBK is aberrantly overexpressed in various cancers, such as gastric^9^, glioma^11^, lung^13^, colon^14^, breast^17^, nasopharyngeal^36^, and prostate cancer^37^, but is barely detected in normal tissues except for fetal and germ cells^7^. Here, our results also showed that PBK expression is strongly upregulated in HGSOC tissues and ovarian cancer cell lines. Previous studies have demonstrated that PBK participates in several key biological processes, including the DNA damage response^38^, cell proliferation^11^, mitosis^39^, migration, and invasion^9,18,19^. Moreover, high PBK expression was associated with a poor prognosis in many cancers. In the current study, we identified that high PBK expression was correlated with unfavorable OS and PFS in all cases of HGSOC, and PBK conferred chemoresistance in vitro and in vivo. Therefore, PBK is a promising molecular target for targeted therapy in ovarian cancer.

Autophagy plays a significant role in the occurrence, development, and treatment of cancer. Although much controversy surrounds the issue, it is commonly believed that autophagy suppresses cancer progression in the initial stage of cancer, and enhanced autophagy promotes cancer survival and proliferation once cancer is established^40,41^. However, the molecular mechanisms in the regulation of ovarian cancer autophagy need to be investigated. Here, we verified for the first time that PBK could promote ovarian cancer cell autophagic flux through activating the mTOR pathway. We further illuminated that PBK activated the mTOR pathway by phosphorylating its substrate ERK1/2. Thus, PBK is a novel autophagy regulator in cancer development. The regulation of autophagy through modulating PBK might be an effective method for ovarian cancer treatment.

Recent studies have suggested that the activation of autophagy is correlated with chemoresistance in lung and liver cancers^42,43^. Wang et al.^44^ reported that autophagy induction contributed to cisplatin resistance through activating the ERK pathway in ovarian cancer cell lines, and autophagy inhibition or ERK knockdown increased cisplatin-induced cell death. Forced expression of PBK induced the resistance to oxaliplatin (OXA) treatment in colorectal cancer^28^. A recent study also showed that PBK promoted the resistance to EGFR tyrosine kinase inhibitors through activating c-Jun in nonsmall cell lung cancer^45^. PBK suppressed the anti-tumor effects of doxorubicin by interacting with p53 and p21 inhibition^31^. These results indicated the strong efficacy of PBK in chemoresistance. In this study, overexpression of PBK attenuated the sensitivity of ovarian cancer cells to cisplatin treatment through inducing autophagy in vitro and in vivo. Furthermore, PBK significantly decreased cisplatin-induced ovarian cancer cell apoptosis. Inhibition of autophagy or the ERK1/2 pathway partially reversed PBK-induced cisplatin resistance and apoptosis. Therefore, targeting PBK might be a prospective strategy to overcome cisplatin resistance.

Targeted kinase inhibitors are the research focus in the targeted therapy of various cancers. The PBK inhibitor HI-TOPK-032 could effectively suppress colon cancer and glioblastoma growth in vitro and in vivo^11,14^. Two other specific PBK inhibitors, OTS514 and OTS964, could significantly decrease PBK expression and inhibit tumor proliferation in lung and kidney cancer and acute myeloid leukemia^46–48^. Ikeda et al.^21^ demonstrated that these two compounds inhibited cell growth in the xenograft model and patient-derived primary ovarian cancer cells, especially in cells derived from malignant ascites samples. In this study, we reported that OTS514 enhanced cisplatin-induced ovarian cancer cell death. The combination of a PBK inhibitor with traditional chemotherapy drugs might be a novel approach for ovarian cancer therapy. However, whether it can be applied clinically needs further investigation.

The abnormal high expression of PBK in cancers is attributed to the deregulation of several transcription factors, such as E2F, ATF, c-Myc, and FOXM1^49–51^. However, the regulatory mechanism of PBK in ovarian cancer remains unclear. EVI1 is overexpressed, and high EVI1 expression indicates a poor patient outcome in ovarian cancer. EVI1 also promotes cell proliferation and migration in ovarian epithelial cells^52,53^. Additionally, high EVI1 expression was observed in drug-resistant patients, suggesting that EVI1 might be a potential predictive marker of chemotherapy resistance in advanced ovarian serous carcinomas^54^. In the current study, we reported that EVI1 could directly bind to the PBK promoter region and promote PBK transcription. High EVI1 expression was correlated with high PBK expression in the patient cohort. Thus, we speculated that EVI1/PBK might be an important axis in promoting the progression of ovarian cancer malignant behavior.

In summary, our study showed that PBK was highly expressed in HGSOC samples and ovarian cancer cell lines. High PBK expression was associated with a poor prognosis, metastasis, and cisplatin resistance in HGSOC. Overexpression of PBK attenuated ovarian cancer cell sensitivity to cisplatin treatment and promoted autophagy flow through the ERK/mTOR axis. We further demonstrated that PBK enhanced cisplatin resistance through activating autophagy in ovarian cancer cell lines and a xenograft model. Additionally, our results verified that EVI1 could promote PBK transcription by directly targeting the PBK promoter region. In conclusion, PBK might be useful for the diagnosis, prognosis, and target therapy of ovarian cancer.

## Materials and methods

### Patients and tissue samples

The retrospective study included 234 cases of HGSOC and 54 cases of normal FT tissues collected at Qilu Hospital of Shandong University from April 2009 to July 2015. The HGSOC specimens were from primary ovarian cancer patients with no previous surgery or chemotherapy. The normal FT tissues were derived from patients who had a benign gynecologic tumor and received hysterectomy and bilateral salpingo-oophorectomy. Ethical approval was obtained from the Ethics Committee of Shandong University. All patients provided written informed consent. The clinical pathologic characteristics of these samples are shown in Table 1.

### Cell lines and cell culture

SKOV3 and OVCAR3 cell lines were purchased from American Type Culture Collection (ATCC, Manassas, VA, USA). A2780, A2780/DDP, and HEY cell lines were kind gifts from Jianjun Wei’s Laboratory. HO8910, HO8910PM, and HEK293T were obtained from the Chinese Academy of Sciences (Shanghai, China). FTE187 was obtained from Jinsong Liu’s Laboratory. A2780, A2780/DDP, HO8910, HO8910PM, and OVCAR3 were cultured in RPMI 1640 supplemented with 10% or 20% fetal bovine serum (FBS); HEK293T, FTE187, and SKOV3 were maintained in Dulbecco’s modified Eagle’s medium (DMEM), M199, and McCoy’s 5A medium, respectively, supplemented with 10% FBS (all from Gibco, Grand Island, NY, USA). All cells were cultured in a humidified incubator at 37 °C with 5% CO_2_.

### Antibodies and reagents

Antibodies for PBK (ab75987), ATG7 (ab52472), LC3B (ab51520), p-ERK1/2 (ab72699), ERK1/2 (ab17942), and EVI1 (ab124934) were from Abcam (Cambridge, UK); β-actin (A5441), CDDP (PHR1624), Mitomycin C (M4287), Baf A1 (196000), and CQ (C6628) were all purchased from Sigma-Aldrich (St. Louis, MO, USA); antibodies for poly (ADP-ribose) polymerase (PARP, 9542S), Caspase-3 (29629S), Cleaved-caspase-3 (9664S), mTOR (2972S), p-mTOR (5536S), p-p70S6K (9206S), p70S6K (9202S), p-4EBP1 (9459S), 4EBP1 (9452S), DYKDDDDK Tag (Flag, 14793S) and Epithelial-Mesenchymal Transition (EMT) Antibody Sampler Kit (9782) were obtained from Cell Signaling Technology (Danvers, MA, USA). U0126 (S1102) and OTS514 (S7652) were purchased from Selleck Chemicals (Houston, TX, USA).

### Western blotting analysis

Cells were lysed in Cell lysis buffer for western and IP (P0013; Beyotime, Shanghai, China) with 1 mM PMSF. After separating proteins by SDS-PAGE and transferring them to PVDF membranes (Merck Millipore, Burlington, MA, USA), the membranes were incubated overnight with primary antibodies, and then with the appropriate horseradish peroxidase-linked secondary antibodies, followed by detection using an enhanced chemiluminescence detection kit (ORT2655; PerkinElmer, Waltham, MA, USA). β-Actin was used as an endogenous control. The relative protein level was analyzed using ImageJ 1.47 (US National Institutes of Health).

### Immunofluorescence assay

Cells were fixed with 4% paraformaldehyde for 15 min, blocked with normal donkey serum for 30 min at room temperature (RT), incubated with the LC3B primary antibody (1:100) overnight at 4 °C, and then incubated with goat anti-rabbit IgG Alexa Fluor-488 (1:200; A-11008; Invitrogen, Waltham, MA, USA) for 1 h at 37 °C in the dark. The nuclei were counterstained with DAPI. Zeiss LSM 780 (Carl Zeiss, Jena, Germany) was used for fluorescence detection.

### RNA interference (RNAi)

Specific siRNA and negative control siRNA were synthesized by GenePharma (Shanghai, China). Cells at the appropriate confluence were transfected with specific siRNA or negative control siRNA for 24 h with Lipofectamine 2000 reagent according to the manufacturer’s protocol (11668-019; Invitrogen), and then the gene silencing efficiency was detected by western blotting. The sequences of siRNAs are shown in Supplementary Table S1.

### Quantitative real-time PCR (qPCR)

Total RNA was extracted using TRIzol reagent (15596018; Invitrogen), and qPCR was performed as described previously^55^. cDNA was synthesized using the PrimeScript RT reagent Kit (RR037A, TaKaRa, Kyoto, Japan). qPCR was conducted using the 7900HT Fast Real Time PCR System (Applied Biosystems, Waltham, MA, USA) with SYBR Premix Ex Taq (RR420A, TakaRa). The mRNA level of specific genes was normalized against β-actin using the comparative Ct method (2^−ΔΔCt^). The primers used are shown in the Supplementary information.

### Flow cytometry assay

Treated cells were collected, rinsed twice with PBS and then resuspended in 1× Binding Buffer (556547; BD Bioscience, Franklin Lakes, NJ, USA). The suspension was incubated with 5 μl of FITC AnnexinV for 25 min and 5 μl of propidium iodide (PI) for 15 min in the dark at RT. FITC or PI-positive cells were detected using a flow cytometer (FC500; Beckman Coulter, Brea, CA, USA). The results were analyzed using FlowJo X 10.0.7 R2 software.

### Tumor formation assay in nude mice

Female athymic BALB/c nude mice (4–5 weeks old; NBRI of Nanjing University, Nanjing, China) were maintained in a pathogen-free facility. Cells were trypsin digested, washed with PBS, and then resuspended in PBS. Next, 200 μl of the suspended cells (1 × 10^7^ cells) were subcutaneously injected into the left armpit of each mouse. The tumor volumes were calculated as the length × width^2^  × 0.5. When the tumor volumes reached 50–200 mm^3^, the mice received intraperitoneal injections or the oral administration of corresponding compounds. The subcutaneous growth of each tumor was measured every 5 days. Twenty-five days post injection, the mice were sacrificed to determine the tumor volumes and were photographed. All the animal experiments were performed with the approval of Shandong University Animal Care and Use Committee.

### Luciferase reporter assay

Cells were transiently co-transfected with PCMV-EVI1, PGL4.26-PBK, and pRL-TK using Lipofectamine 2000 for at least 24 h. Luciferase activity was measured using the Dual-Glo Luciferase Assay System (E2920; Promega, Fitchburg, WI, USA) following the manufacturer’s instructions. The relative luciferase activity was determined by the ratio between firefly luminescence and renilla luminescence.

### Statistical analysis

All experiments were repeated at least three times independently. The data were expressed as the means ± SEM and were analyzed by one-way ANOVA using SPSS v17.0 (SPSS Inc., Chicago, IL, USA). Chi-squared test was used to analyze the differences in clinical characteristics. OS analysis was performed by Kaplan–Meier and log-rank tests. Images were processed using GraphPad Prism 7.00 (GraphPad Software, La Jolla, CA, USA) and Adobe Photoshop CC 14.0 (Adobe, San Jose, CA, USA). *P* < 0.05 was considered statistically significant.

## Supplementary information

revised Supplementary Information
