# Supplementary material for: PBK, targeted by EVI1, promotes metastasis and confers cisplatin resistance through inducing autophagy in high-grade serous ovarian carcinoma
Source: Cell Death Dis. 2019 Feb 18;10(3):166. doi: 10.1038/s41419-019-1415-6 (PMC6379381; doi:10.1038/s41419-019-1415-6)
Supplement: Supplementary file 1 — revised Supplementary Information [file 41419_2019_1415_MOESM1_ESM.docx]

**Supplementary Materials and Methods**

**Plasmid construction and lentivirus production**

The PCMV-PBK, PCMV-ATG7, and PCMV-EVI1 plasmids were generated by inserting the open reading frame of corresponding genes into pLenti-C-Myc-DDK-IRES-Puro vector (PCMV, PS100069, OriGene, Rockville, MD, USA). pLKO.1 PBK shRNA vector (TRCN0000001806) was purchased from Sigma-Aldrich. The promotor of PBK was cloned into pGL4.26 (E8441, Promega) to generate the wild-type (WT) construct. Overlap extension PCR was used to generate mutant-type (MT) plasmid. All the constructs were confirmed by DNA sequencing. Plasmid transient transfection was performed by use of Lipofectamine 2000. Lentivirus particles were produced in HEK293T cells packaged by psPAX2 and pMD2.G. To obtain stable transfection cell lines, cells were infected with lentivirus for 24 h and then selected for two weeks in corresponding medium containing 2 μg/ml puromycin (P8833, Sigma-Aldrich).

**Immunohistochemistry**

Formalin-fixed and paraffin-embedded tissues were sectioned at 4 μm per slide. Tissue slides were incubated at 70°C for 30 min and then deparaffinized in xylene immediately. After rehydration in a graded series of ethanol, tissue slides were submerged for antigen retrieval in citrate buffer (pH=6.0) using a microwave oven. The slides were incubated with primary antibody overnight at 4°C, then incubated with corresponding secondary antibodies, stained with diaminobenzidine (DAB; ZSGB-BIO, Beijing, China) and counterstained with hematoxylin. The high or low expression of each sample was decided by two pathologists based on the intensity and extent of staining across the sections. The intensity of staining was scored as 0 (negative), 1 (weak), 2 (moderate), or 3 (strong). The extent of staining was based on the percentage of positive tumor cells: 1 (0–25%), 2 (26–50%), 3 (51–75%), and 4 (76–100%). The final score of each sample was the average score of the two duplicates. Low expression means the final score was 0–4 and high expression means the final score was 5–12, respectively. Cisplatin resistance or sensitivity was defined as the presence or absence of tumor relapse progression within 6 months from the end of primary platinum-based chemotherapy.

**High-throughput differential gene expression analysis**

The high-throughput mRNA-Seq experiments were conducted by Berry Genomics (Beijing, China). mRNA-seq library was prepared for sequencing following standard Illumina protocols. In brief, total RNAs from Ctr or si PBK cells were extracted using TRIzol reagent and treated with RNase-free DNase I (M0303S, NEB, Ipswich, MA, USA) to remove genomic DNA. mRNA was extracted with Dynabeads oligo(dT) (Invitrogen). Double-stranded complementary DNAs are synthesized using Superscript II reverse transcriptase (Invitrogen) and random hexamer primers. The cDNAs were then fragmented by nebulization and the standard Illumina protocol was followed thereafter, to create the mRNA-seq library. For the data analysis, basecalls were performed using CASAVA. Reads were aligned to the genome using the split read aligner TopHat (v2.0.7) and Bowtie 2, using default parameters. HTSeq was used for estimating their abundances.

**Migration and** **invasion assays**

Migration assay was conducted in 24-well transwell chambers system with 8 µm pores (353097, BD Biosciences). A total of 1-2×10^5^ cells were added into the upper chambers in 200 μl serum-free medium, and lower chambers were filled with 700 μl culture media containing 20% FBS. After culturing for appropriate time, successfully invaded cells were fixed with methanol, stained with crystal violet and counted under a light microscope. The invasion assay was performed in the same manner as the migration assay described above, except that the top chamber was coated with matrigel (354234, BD Biosciences).

**Cell proliferation assay**

Cells were seeded onto 96-well plates, then treated with 0.1% DMSO (as control) or the corresponding compounds for indicated time. Cell proliferation was measured by use of Cell Counting Kit-8 (CCK8) assay. 10 μl CCK8 solution was added to each well and continued to incubate for 2 h at 37°C. Absorption was measured at the wavelength of 540 nm using a microplate reader (Thermo Scientific, Waltham, MA, USA).

**Clonogenic assay**

Cells were seeded in a six-well plate (800-1000 cells/well) and cultured for 10-14 days. The colonies were fixed with methanol for 15 min, stained with 0.5% crystal violet for 15 min, and the number of colonies containing > 50 cells was counted.

**Chromatin immunoprecipitation (CHIP) assay**

CHIP assay was performed with use of EZ-ChIP Chromatin immunoprecipitation kit following the manufacturer's instructions (17-371, Merck Millipore). In brief, A2780 cells transfected with PCMV-EVI1 were cross-linked using 37% formaldehyde, washed with cold 1×PBS and collected into 1.5 ml EP tubes. The cells were then lysed in lysis buffer and sonication was executed to shear the DNA to 200-1000 bp fragments. 10 µl supernatants were retained as input control. The DNA-protein complexes were then immunoprecipitated by Flag antibody or negative control IgG. After the cross-links were reversed, the associated DNA fragments were eluted, which was followed by analysis of the resultant DNA by RT-PCR.

**Supplementary Figures and Figure legends:**


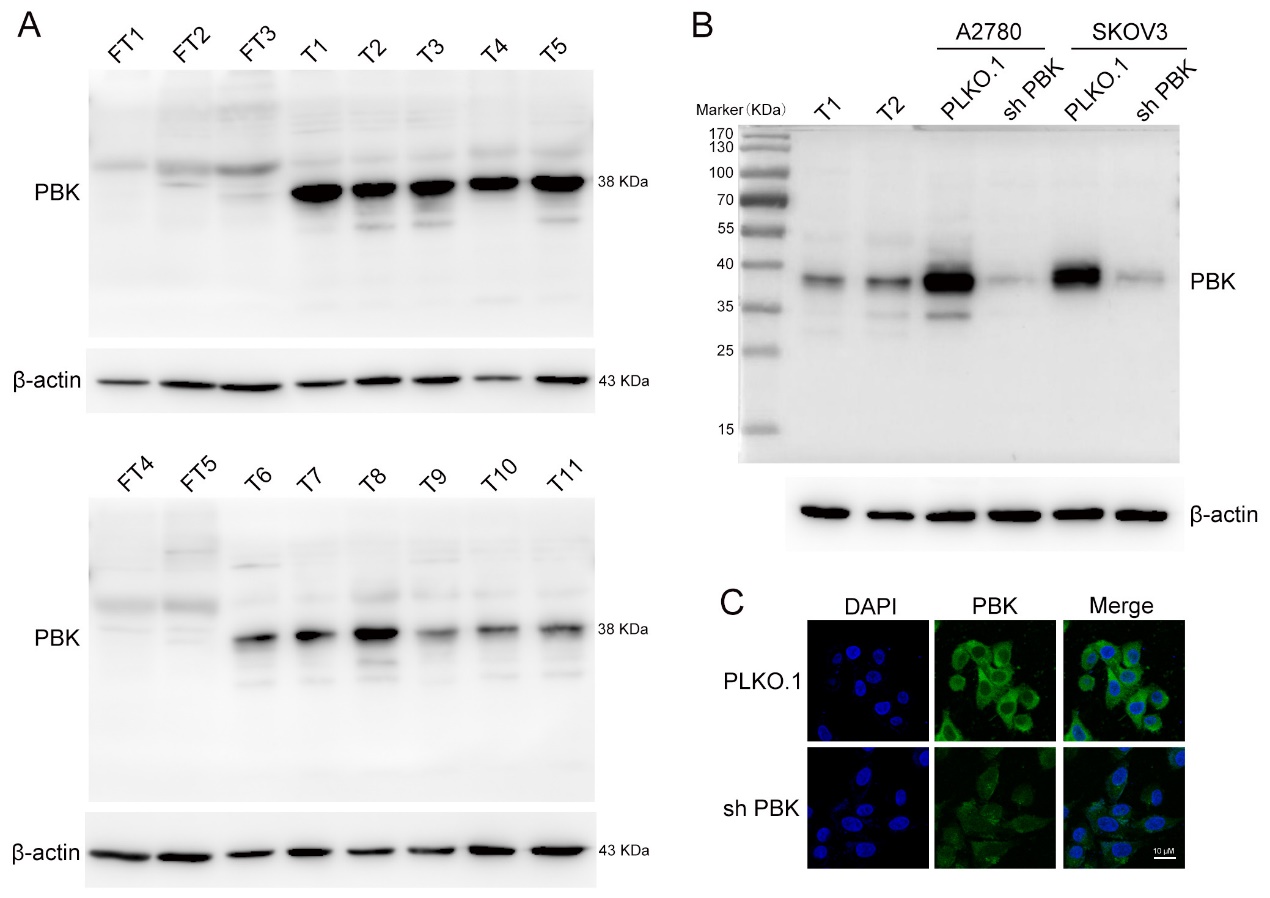


Supplementary Figure S1. (A) Uncut western blot bands in Figure 1B. (B) Western blot analysis of the specificity of PBK antibody used in HGSOC tissues (T1-T2), A2780 and SKOV3 cells transfected with PLKO.1 or PBK shRNA (sh PBK). (C) Immunofluorescence images of PBK in A2780 cells transfected with PLKO.1 and PBK shRNA (sh PBK). DAPI staining of nuclei was in blue. Scale bar: 10 μm.

**
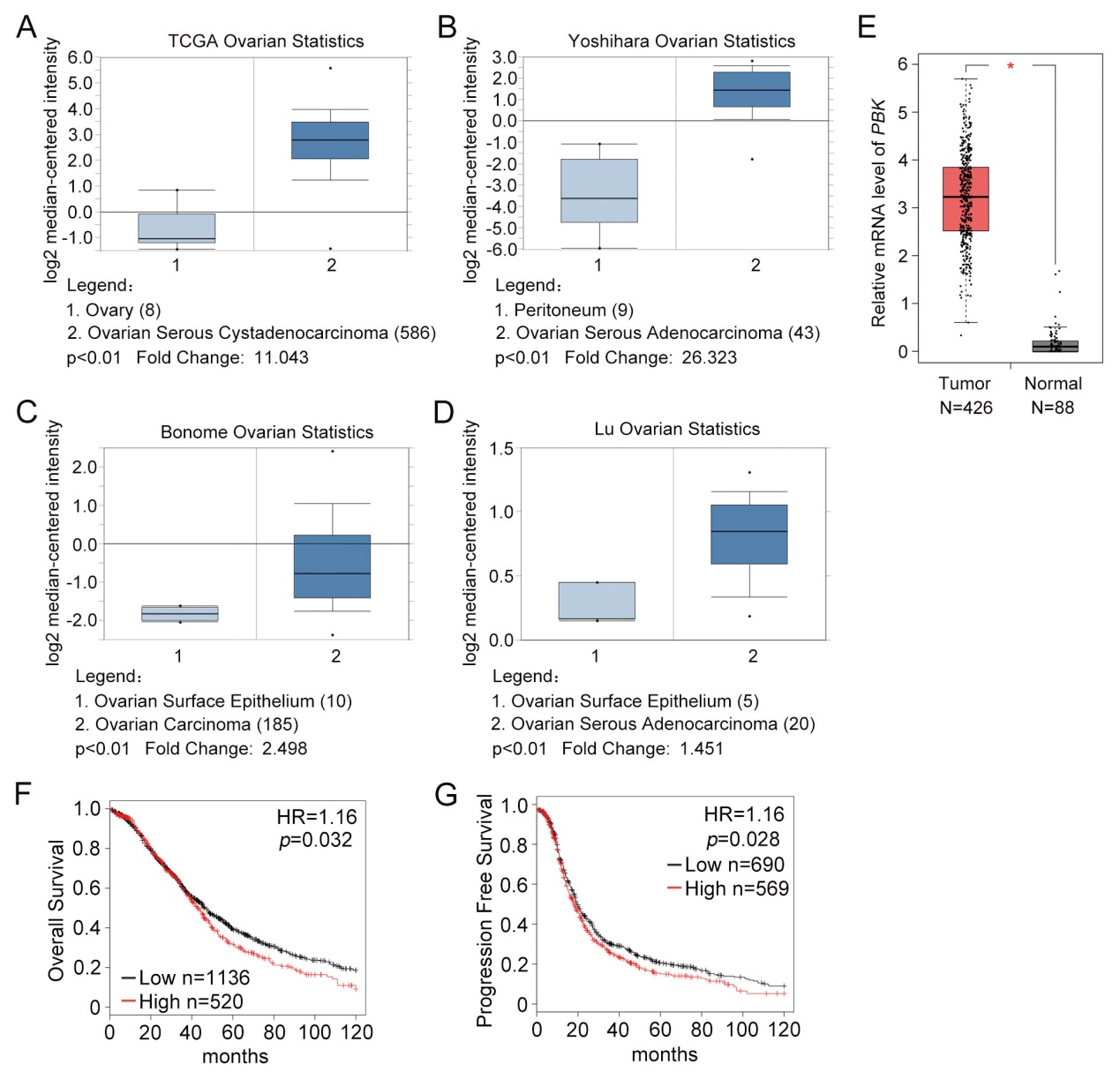
**

Supplementary Figure S2. PBK was overexpressed and associated with poor overall survival (OS) and progress progression-free survival (PFS) in ovarian cancer. (A-E) Oncomine (https://www.oncomine.org) and GEPIA (http://gepia.cancer-pku.cn/) datasets showed that the expression of PBK was relatively higher in ovarian cancer compared with normal controls. OS (F) and PFS (G) of PBK in ovarian cancer using microarray data from Kaplan Meier-plotter.


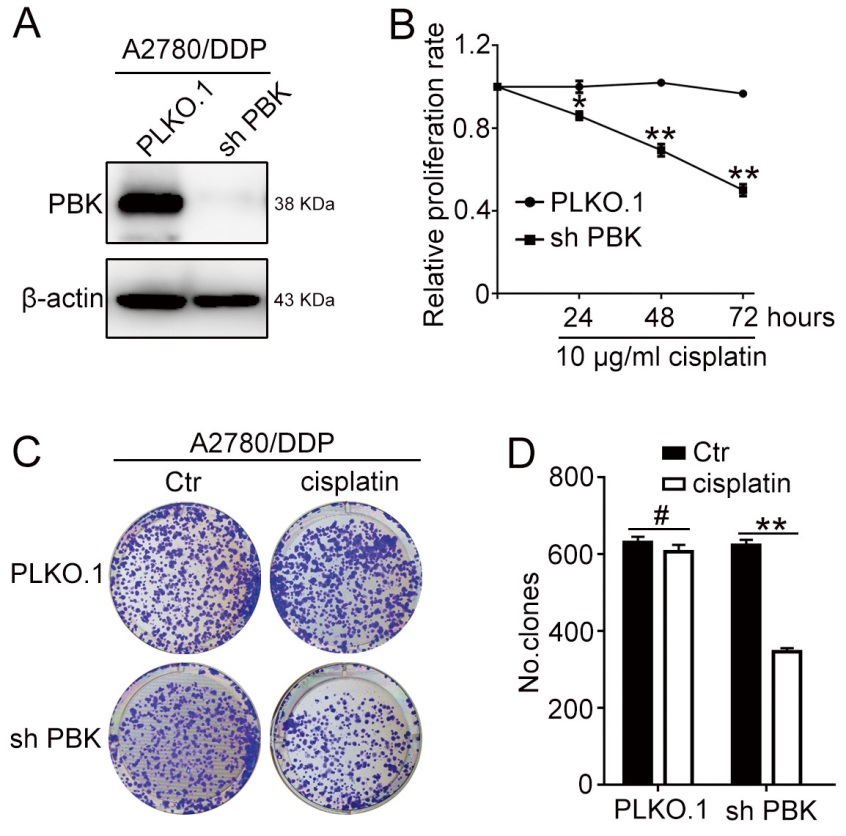


Supplementary Figure S3. PBK conferred chemoresistance in cisplatin-resistant A2780/DDP cells. A2780/DDP cells were stably transfected with PLKO.1 or PBK shRNA (sh PBK), then treated with 10 μg/ml cisplatin (CDDP) for 24, 48 and 72 h. (A) Protein levels of PBK and β-actin were analyzed by western blot. (B) Relative proliferation rate was measured using Cell Counting Kit-8 (CCK8). (C) Clonogenic assay was performed to assess the colony formation efficiency in A2780/DDP cells stably transfected with PLKO.1 and PBK shRNA with or without 10 μg/ml cisplatin treatment. (D) Quantification of the number of clones in C. (Data are mean ± SEM, ^#^*p* > 0.05, **p* < 0.05, ***p* < 0.01, n = 3).


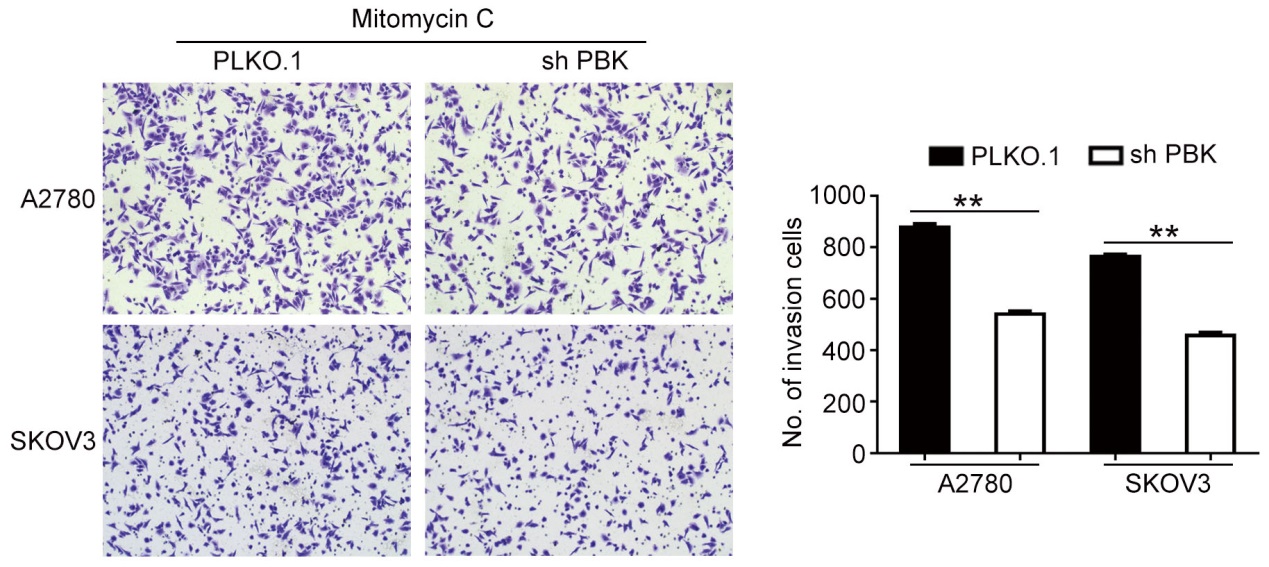


Supplementary Figure S4. Transwell assay was performed to determinate the effects of PBK knockdown on the invasion of A2780 and SKOV3 cells. Cells were pre-treated with mitomycin C (10 μg/ml) for 2 hours to exclude the influence of cell proliferation before plating them into the transwells. (Data are mean ± SEM, ***p* < 0.01, n = 3).


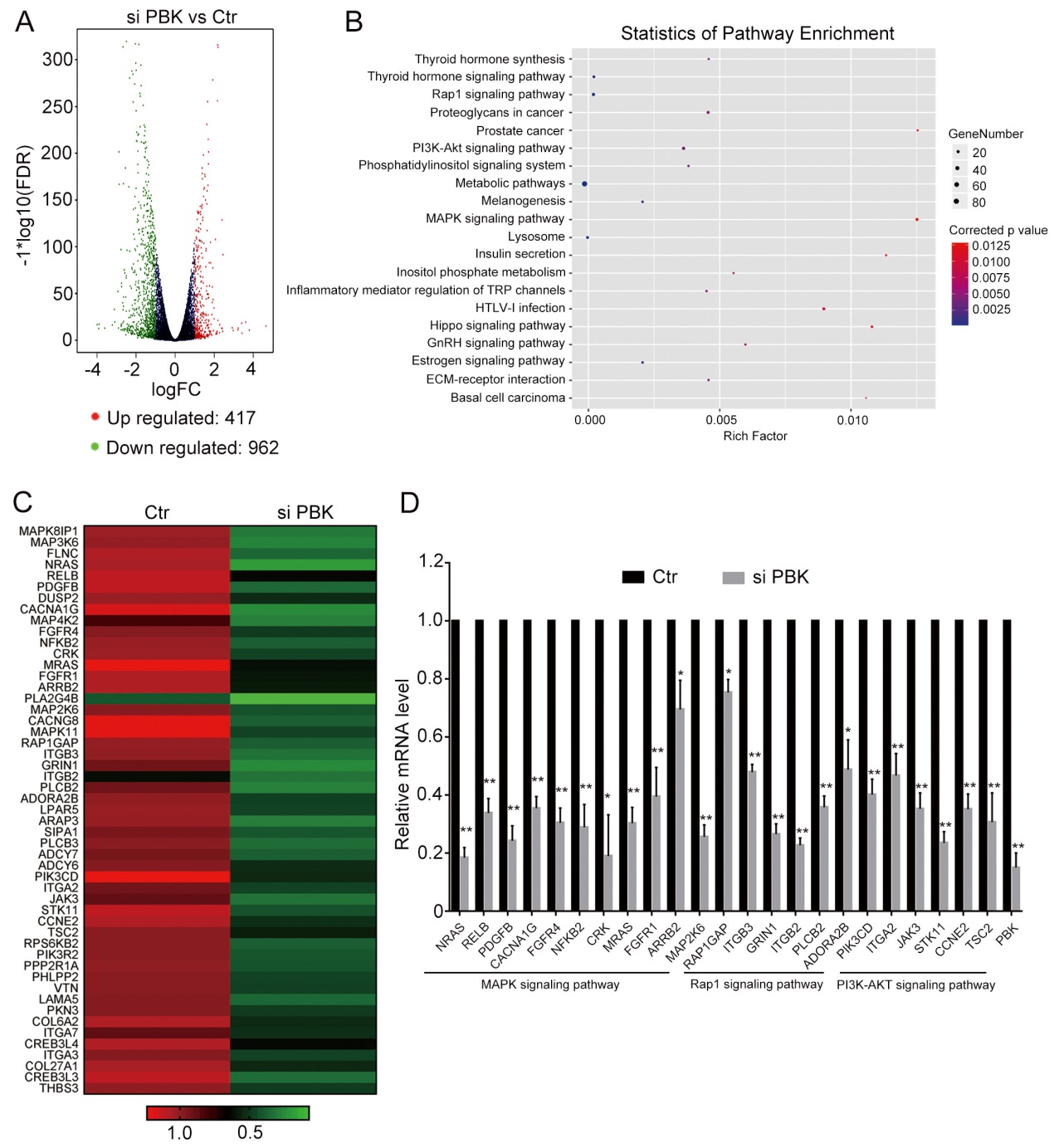


Supplementary Figure S5. NGS analysis of PBK influenced signal pathways. High-throughput RNA Sequencing analysis was conducted to determine the mRNA expression profile of A2780 cells transfected with PBK siRNA (si PBK) or negative control siRNA (Ctr) for 48 h. (A) Volcano plot exhibited the differentially expressed genes (DEGs) in si PBK and Ctr groups. (B) Pathway analysis of DEGs based on the KEGG database. (C) Heatmap of DEGs in MAPK, Rap1 and PI3K-AKT signaling pathway between si PBK and Ctr groups. (D) qPCR verification of representative DEGs between si PBK and Ctr group in A2780 cells. (Data are mean ± SEM, **p* < 0.05, ***p* < 0.01, n = 3).


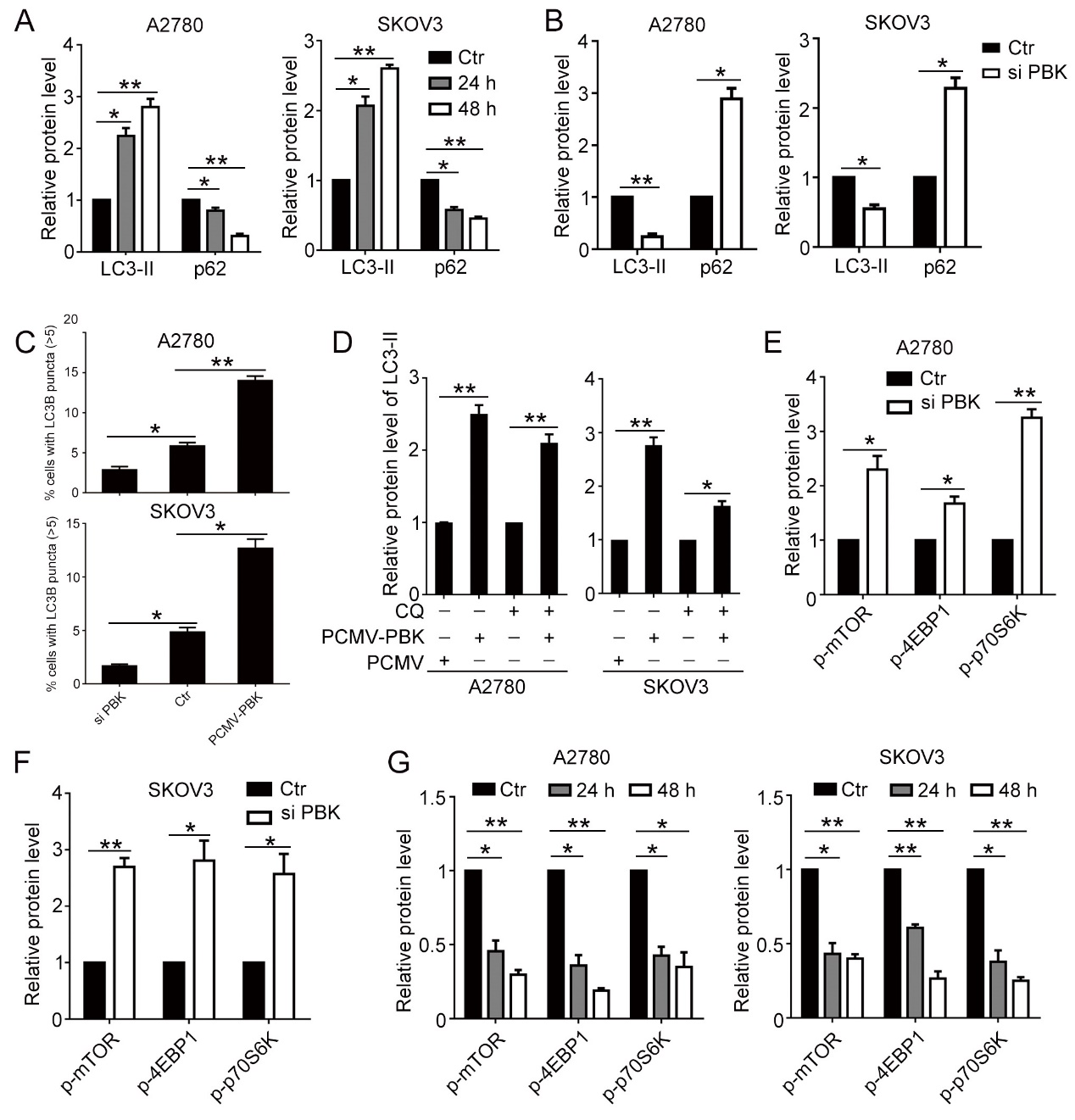


Supplementary Figure S6. (C) The proportion of cells containing LC3B puncta (> 5) in Figure 5C. Other figures showed the quantification of relative protein expression levels in Figure 4A-4F. (Data are mean ± SEM, **p* < 0.05, ***p* < 0.01, n = 3).


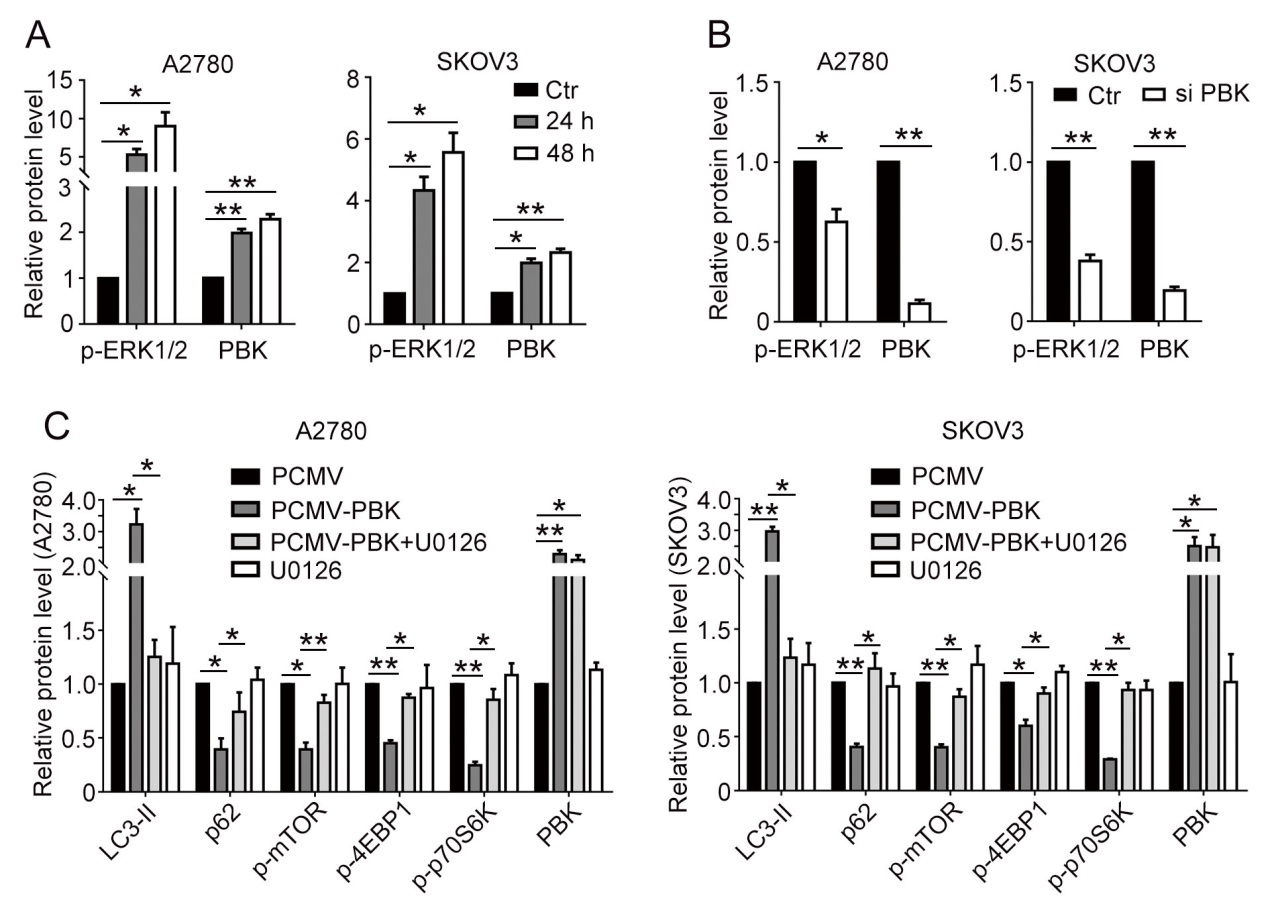


Supplementary Figure S7. Quantification of relative protein expression levels in Figure 4G-4I. (Data are mean ± SEM, **p* < 0.05, ***p* < 0.01, n = 3).


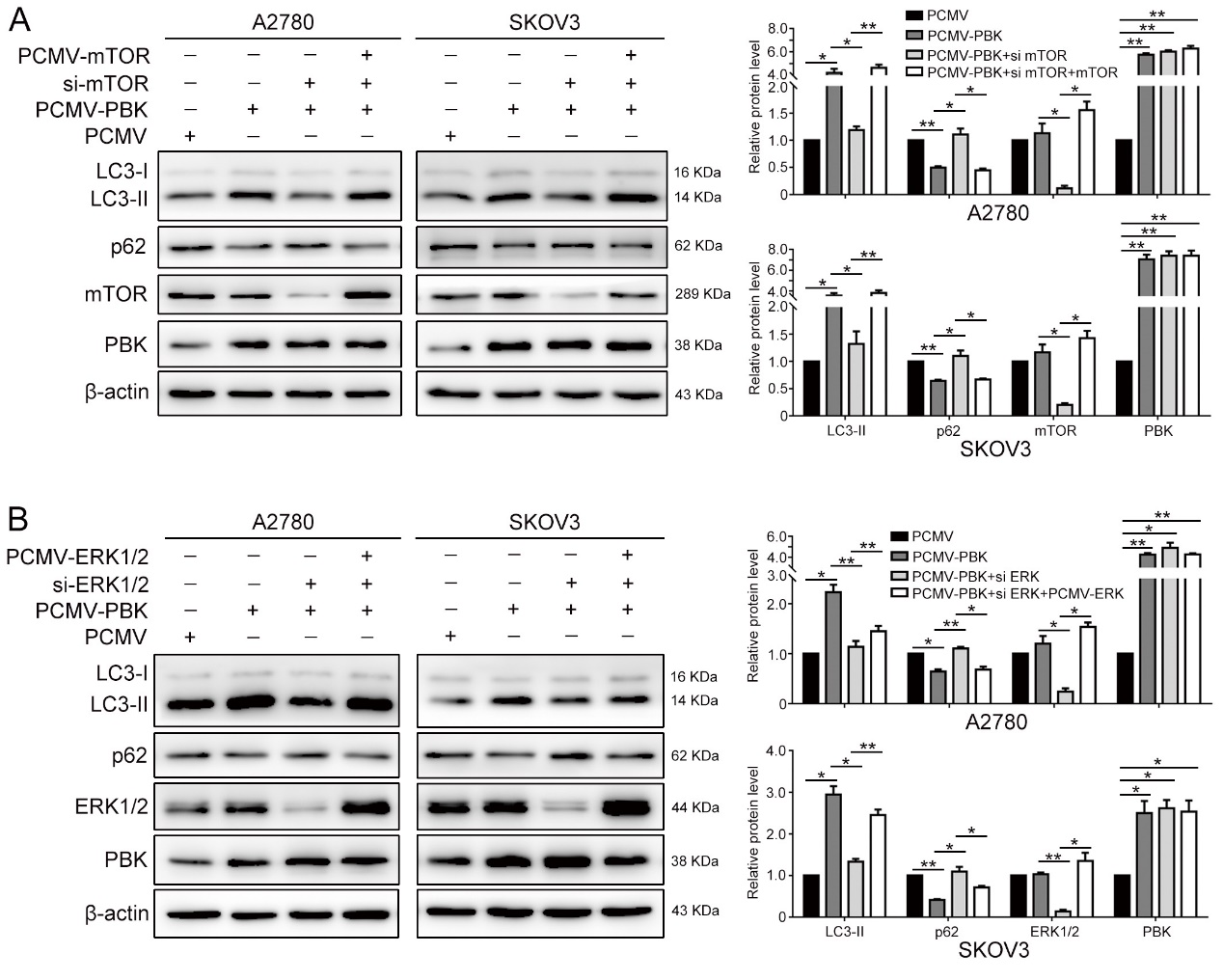


Supplementary Figure S8. PBK promoted autophagy through ERK/mTOR axis in A2780 and SKOV3 cells. (A) Western blot analysis of protein levels of LC3-I, LC3-II, p62, mTOR, PBK and β-actin in A2780 and SKOV3 cells transfected with PCMV, PCMV-PBK, PCMV-mTOR or mTOR siRNA (si mTOR) for 48 h. (B) Western blot analysis of protein levels of LC3-I, LC3-II, p62, ERK1/2, PBK and β-actin in A2780 and SKOV3 cells transfected with PCMV, PCMV-PBK, PCMV-ERK1/2 or ERK1/2 siRNA (si ERK1/2) for 48 h. . (Data are mean ± SEM, **p* < 0.05, ***p* < 0.01, n = 3).


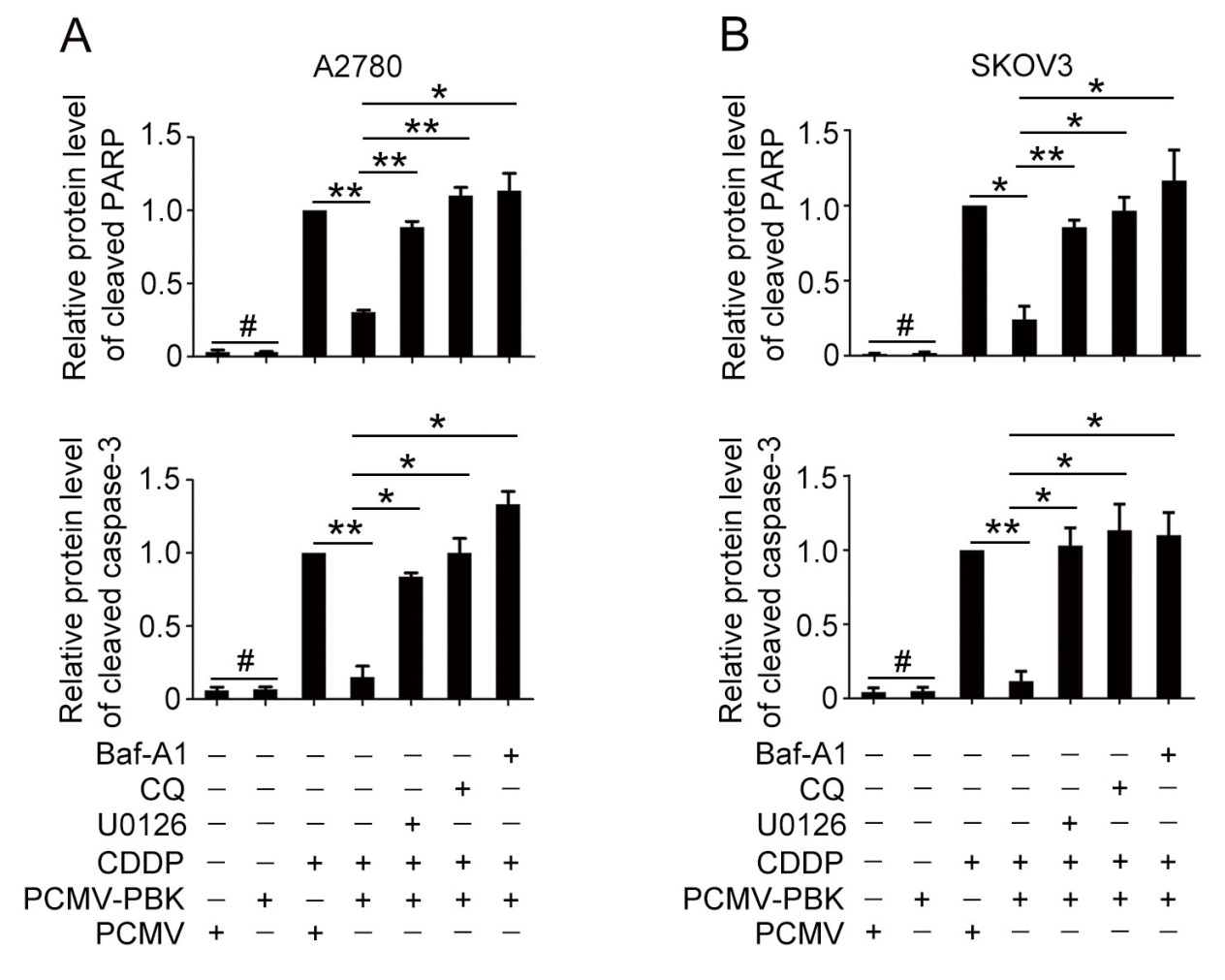


Supplementary Figure S9. Quantification of relative protein expression levels of cleaved PARP and cleaved caspase-3 in Figure 5B. (Data are mean ± SEM, ^#^*p* > 0.05, **p* < 0.05, ***p* < 0.01, n = 3).


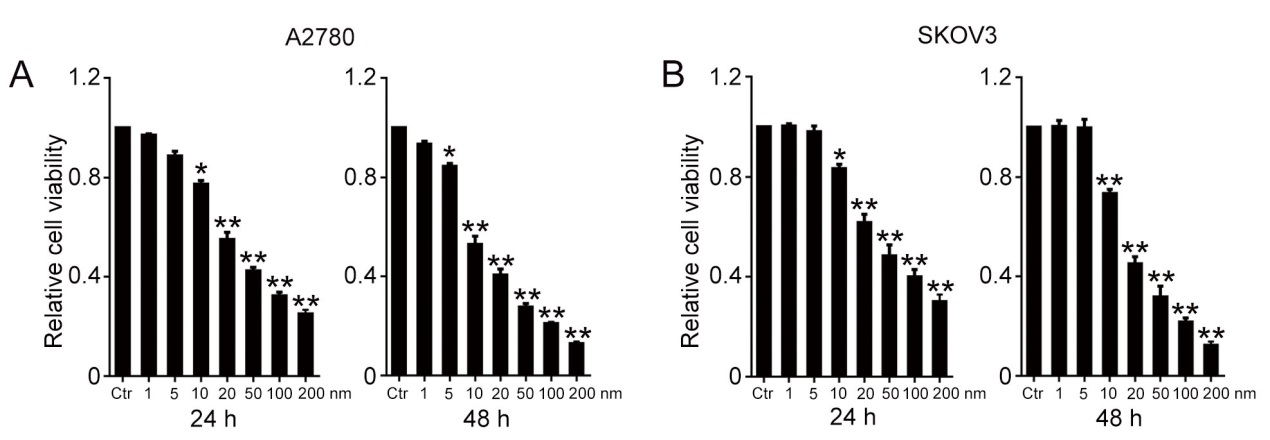


Supplementary Figure S10. CCK8 assay determination of relative cell viability in A2780 (A) and SKOV3 (B) cells treated with 1, 5, 10, 20, 50, 100, 200 nm OTS514 for 24 h and 48 h. (Data are mean ± SEM, **p* < 0.05, ***p* < 0.01, n = 3).


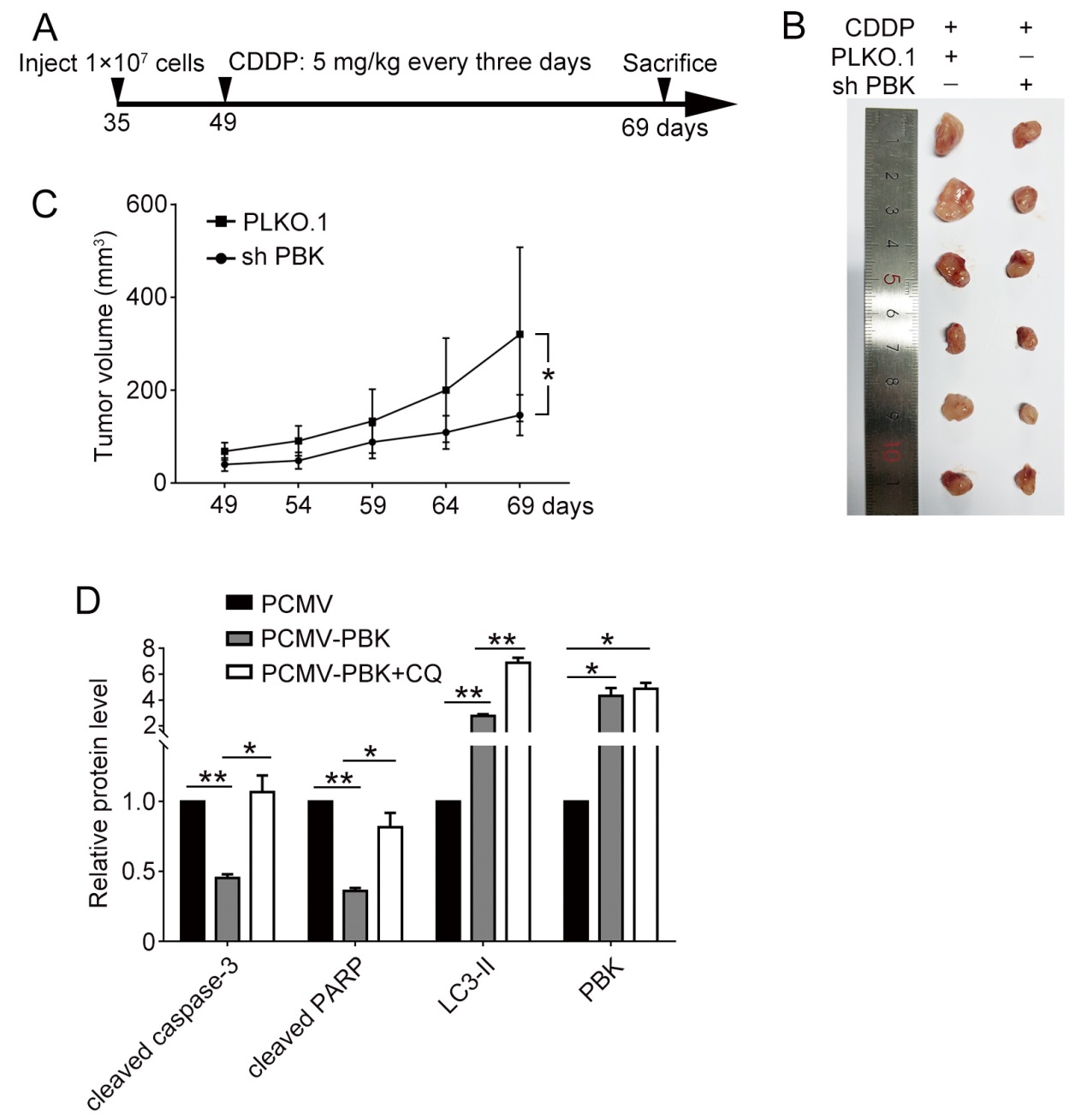


Supplementary Figure S11. (A) Experimental design of experimental protocols in BALB/c nude mice. 35 days old mice were subcutaneously injected with cells stably transfected with PLKO.1 or PBK shRNA (sh PBK). When the tumor volumes reached 50-200 mm^3^ at day 49, tumor bearing mice then received intraperitoneal injection of CDDP (5 mg/kg, every three days). The volumes of tumors were measured every five days using a vernier caliper. 20 days after injection, the mice were sacrificed to determine tumor volumes and and were photographed. (B) Tumors from each group were shown. (C) The tumor volumes of each group. (D) Quantification of relative protein expression levels of cleaved caspase-3, cleaved PARP, LC3-II, and PBK in Figure 7D. (Data are mean ± SEM, **p* < 0.05, ***p* <0.01, n = 6).

**Supplementary Table S1. Primer sequences used for PCR. siRNA sequences were also shown.**

| **siRNA sequences** | |
| --- | --- |
|  | **Sequences (5' to 3')** |
| PBK-siRNA | CCCUGAGGCUUGUUACAUU |
| EVI1-siRNA | UCUAAGGCUGAACUAGCAG |
| ERK1/2-siRNA | GACCGGAUGUUAACCUUUAUU |
| ATG7-siRNA | GGAGUCACAGCUCUUCCUU |
| mTOR-siRNA | CUUCGAGACAUGAGUCAGCUU |
| **Primer sequences used for ChIP-PCR** | |
| PBK-promoter1-F | TTGGAGCTGTTAAGCAAT |
| PBK-promoter1-R | AGAATATGAGCCAAGACGT |
| PBK-promoter2-F | TACCTCGAAACAGAAATGAG |
| PBK-promoter2-R | GTGCTGAAGGCTGAAGCT |
| **Primer sequences used for qPCR** | |
| **Gene** | **Primer sequences (5' to 3')** |
| β-actin-F | CATGTACGTTGCTATCCAGGC |
| β-actin-R | CTCCTTAATGTCACGCACGAT |
| PBK-F | GAAGAGGACTGAGAGTGGCT |
| PBK-R | CTTCTGCATAAACGGAGAGGC |
| EVI1-F | TTGCCAAGTAACAG-CTTTGCTG |
| EVI1-R | CCAAAGGGTCCGAATGTGACTT |
| NRAS-F | GAAACCTCAGCCAAGACCAGAC |
| NRAS-R | GGCAATCCCATACAACCCTGAG |
| RELB-F | TGTGGTGAGGATCTGCTTCCAG |
| RELB-R | TCGGCAAATCCGCAGCTCTGAT |
| PDGFB-F | GAGATGCTGAGTGACCACTCGA |
| PDGFB-R | GTCATGTTCAGGTCCAACTCGG |
| CACNA1G-F | TTCACCGCAGTCTTTCTGGCTG |
| CACNA1G-R | TGACGGAGATGAGCACCAACAG |
| FGFR4-F | AACACCGTCAAGTTCCGCTGTC |
| FGFR4-R | CATCACGAGACTCCAGTGCTGA |
| NFKB2-F | GGCAGACCAGTGTCATTGAGCA |
| NFKB2-R | CAGCAGAAAGCTCACCACACTC |
| CRK-F | CCAATGCCTACGACAAGACAGC |
| CRK-R | TGGGAAGTGACCTCGTTTGCCA |
| MRAS-F | CCACCATTGAAGACTCCTACCTG |
| MRAS-R | ACGGAGTAGACGATGAGGAAGC |
| FGFR1-F | GCACATCCAGTGGCTAAAGCAC |
| FGFR1-R | AGCACCTCCATCTCTTTGTCGG |
| ARRB2-F | CTGACTACCTGAAGGACCGCAA |
| ARRB2-R | GTGGCGATGAACAGGTCTTTGC |
| MAP2K6-F | GGCTACTTGGTGGACTCTGTTG |
| MAP2K6-R | CATCGTGATGCCCAGACTCCAA |
| RAP1GAP-F | CCGACATGATCGCGTCCAACTT |
| RAP1GAP-R | GGGTCCAAAGAAGGGCACATCA |
| ITGB3-F | CATGGATTCCAGCAATGTCCTCC |
| ITGB3-R | TTGAGGCAGGTGGCATTGAAGG |
| GRIN1-F | CCAGTCAAGAAGGTGATCTGCAC |
| GRIN1-R | TTCATGGTCCGTGCCAGCTTGA |
| ITGB2-F | AGTCACCTACGACTCCTTCTGC |
| ITGB2-R | CAAACGACTGCTCCTGGATGCA |
| PLCB2-F | CCTGGAAGTGACGGCTTATGAG |
| PLCB2-R | GCTCTGTGAAGGACGAGATGAC |
| ADORA2B-F | GCTCCATCTTCAGCCTTCTGGC |
| ADORA2B-R | AAGGACCCAGAGGACAGCAATG |
| PIK3CD-F | TGCCAAACCACCTCCCATTCCT |
| PIK3CD-R | CATCTCGTTGCCGTGGAAAAGC |
| ITGA2-F | TTGCGTGTGGACATCAGTCTGG |
| ITGA2-R | GCTGGTATTTGTCGGACATCTAG |
| JAK3-F | AGTGACCCTCACTTCCTGCTGT |
| JAK3-R | GGCTGAACCAAGGATGATGTGG |
| STK11-F | CTACTGAGGAGGTTACGGCACA |
| STK11-R | ACGCTGTCCAGCATTTCCTGCA |
| CCNE2-F | CTTACGTCACTGATGGTGCTTGC |
| CCNE2-R | CTTGGAGAAAGAGATTTAGCCAGG |
| TSC2-F | GCACCTCTACAGGAACTTTGCC |
| TSC2-R | GCACCTGATGAACCACATGGCT |
